# Ancient mitogenomics clarifies radiation of extinct Mascarene giant tortoises (*Cylindraspis* spp.)

**DOI:** 10.1038/s41598-019-54019-y

**Published:** 2019-11-25

**Authors:** Christian Kehlmaier, Eva Graciá, Patrick D. Campbell, Margaretha D. Hofmeyr, Silke Schweiger, Albert Martínez-Silvestre, Walter Joyce, Uwe Fritz

**Affiliations:** 1 Museum of Zoology, Senckenberg Dresden, A. B. Meyer Building, 01109 Dresden, Germany; 2 Ecology Area, Department of Applied Biology, Miguel Hernández University, Av. de la Universidad, Torreblanca, 03202 Elche Spain; 3 Department of Life Sciences, Darwin Centre (DC1), Natural History Museum, London, SW7 5BD England UK; 4 Chelonian Biodiversity and Conservation, Department of Biodiversity and Conservation Biology, University of the Western Cape, Bellville, 7535 South Africa; 5 Herpetological Collection, Natural History Museum Vienna, Burgring 7, 1010 Vienna, Austria; 6 Catalonian Reptile and Amphibian Rescue Center (CRARC), 08783 Masquefa, Barcelona Spain; 7 Department of Geosciences, University of Fribourg, 1700 Fribourg, Switzerland

**Keywords:** Palaeontology, Phylogenetics, Herpetology

## Abstract

The five extinct giant tortoises of the genus *Cylindraspis* belong to the most iconic species of the enigmatic fauna of the Mascarene Islands that went largely extinct after the discovery of the islands. To resolve the phylogeny and biogeography of *Cylindraspis*, we analysed a data set of 45 mitogenomes that includes all lineages of extant tortoises and eight near-complete sequences of all Mascarene species extracted from historic and subfossil material. *Cylindraspis* is an ancient lineage that diverged as early as the late Eocene. Diversification of *Cylindraspis* commenced in the mid-Oligocene, long before the formation of the Mascarene Islands. This rejects any notion suggesting that the group either arrived from nearby or distant continents over the course of the last millions of years or had even been translocated to the islands by humans. Instead, *Cylindraspis* likely originated on now submerged islands of the Réunion Hotspot and utilized these to island hop to reach the Mascarenes. The final diversification took place both before and after the arrival on the Mascarenes. With *Cylindraspis* a deeply divergent clade of tortoises became extinct that evolved long before the dodo or the Rodrigues solitaire, two other charismatic species of the lost Mascarene fauna.

## Introduction

The Mascarenes are a group of islands in the Indian Ocean approximately 500 km east of Madagascar that are renowned for their largely lost fauna that rapidly disappeared after the arrival of European settlers in the mid-17^th^ century. Besides the dodo (*Raphus cucullatus*) and the Rodrigues solitaire (*Pezophaps solitaria*), the extinct giant tortoises of the genus *Cylindraspis* belong to the most charismatic representatives of this perished island-endemic fauna. Extinction of the five *Cylindraspis* species most likely occurred between 1735 and 1840, suggesting that they vanished some 100–200 years after the colonization of the Mascarenes and survived the dodo by some 200 years^1–3^. Despite their small size of only approximately 104 km^2^ (Rodrigues), 1,865 km^2^ (Mauritius), and 2,511 km^2^ (Réunion), two islands harboured two giant tortoise species each. On Mauritius, a species with a domed shell (*C*. *inepta*) occurred alongside a species with a flat shell (*C*. *triserrata*). Each reached a straight carapace length of 60–70 cm, exceptionally up to 100 cm. Another domed but small species (*C*. *peltastes*; up to 46 cm) plus a large saddle-backed species (*C*. *vosmaeri*; up to 110 cm) lived on Rodrigues. The largest island, Réunion, was inhabited by only one large-sized species (*C*. *indica*; up to 120 cm)^4^. Recently, it has been proposed that the Mascarene giant tortoises were introduced by early Austronesian sailors^5^, spurring a series of hefty rebuttals^6–9^. Short mitochondrial DNA (mtDNA) sequences of approximately 400 bp length produced in a pioneering ancient DNA (aDNA) study 20 years ago^10^ played a key role in the arguments against the human-mediated arrival of giant tortoises, as this genetic data set clearly shows the distinctiveness of Mascarene tortoises. However, the authenticity of the aDNA sequences for one species (*C*. *triserrata*) was doubted as it clustered in a re-analysis with South American tortoise species^9^. The recent debate underlines that the geographic origin and the radiation of *Cylindraspis* within the Mascarenes are still poorly understood.

The recent extinction of the *Cylindraspis* species make them a relatively easy target for aDNA studies, and since the pioneering study by Austin & Arnold^10^, refined methodologies and NGS technology^11^ allow for better results that have the potential to shed light on the evolution and biogeography of the Mascarene giant tortoises. Using cutting-edge aDNA approaches^12,13^, we here publish near-complete mitochondrial genomes for all five *Cylindraspis* species, with up to three mitogenomes per species and additional, more fragmentary, sequence data for more individuals (Supplementary Information). We analyse our data together with mitogenomes representing all 17 extant tortoise genera, including an extinct species from the Bahamas^14^. In addition, our data set contains newly generated and previously published mitogenomes of all five extant tortoise species from Madagascar and Aldabra and of representatives of all species groups of the tortoise subfamily Testudininae to which *Cylindraspis* belongs^9,15^. Based on this comprehensive data set, we conduct phylogenetic reconstructions, fossil-calibrated molecular clock calculations, and ancestral range analyses. Our study clarifies the phylogenetic and biogeographic origin of *Cylindraspis*, the relationships of its species, and contributes to a better understanding of the lost fauna of the Mascarenes.

## Materials and Methods

### Studied specimens

Eighteen subfossil bone samples and one historic *Cylindraspis* specimen from the Natural History Museums in London (NHM[UK]) and Vienna (NMW) were studied, representing all five species of Mascarene giant tortoises (Table S1). The historic specimen (NMW 1461), a shell of *C*. *vosmaeri* with epidermal scutes, was collected during the late 18^th^ century, prior to the extinction of this species. In addition, fresh samples of 16 extant tortoise species were studied to achieve a broad taxonomic coverage of Testudinidae (for details, see Table S2). Many fresh blood samples originate from tortoises kept in the Zoological Gardens of Dresden, Leipzig, and Vienna or from the captive colony of *Astrochelys yniphora* on Rodrigues. Captive tortoises were sampled for clearing health issues by veterinarians following all relevant guidelines, and samples were later shared with us for this study. Other samples were obtained during fieldwork in South Africa and Namibia from wild tortoises under permits from CapeNature (AAA007-00168-0056), the Limpopo Provincial Government (ZA/Lp/80202), the Department of Environment and Nature Conservation, Northern Cape (245/2015, 246/2015), and the Ministry of Environment and Tourism, Namibia (1420/2009). Wild tortoises were released at the collection site after taking blood samples in accordance with all relevant guidelines, regulations, and methods approved by the Ethics Committee of the University of the Western Cape (ethical clearance number ScRiRC2008/39). Fresh samples were ethanol-preserved and stored at −80 °C until processing.

### Processing of historic and subfossil *Cylindraspis* samples

The 18 subfossil bone specimens were sampled using a Dremel drill equipped either with a core sampling bit or diamond circular saw bit, depending on the specimen. The tool was cleaned with DNA decontamination reagent (Sigma-Aldrich) before reuse. From the historic specimen, dried tissue was sampled from inside the shell. Subsequent sample processing was carried out in the clean room facility of the Senckenberg Natural History Collections Dresden according to established guidelines^16^. Negative controls (water blanks) were included during DNA extraction and library preparation and screened for evidence of contamination.

DNA of the historic tissue sample was extracted using the DNeasy Blood & Tissue Kit (Qiagen). Bone fragments were crushed in a bone mill (Retsch MM400) for 10 sec at 20× /min or in a porcelain mortar by hand. Bone powder was processed according to a protocol optimised for the recovery of short DNA fragments^12^. For each extraction, 50–100 mg of buffer-soaked bone powder was used (Table S3). Then, 4.8–44.4 ng of DNA was converted into single-indexed single-stranded Illumina sequencing libraries according to Gansauge & Meyer^13^ and Korlević *et al*.^17^, including the removal of uracil residues by uracil-DNA glycosylase (UDG) treatment. An initial assessment of DNA preservation and contamination was made by low-level shotgun sequencing of sample NHM(UK) 2000.49 on an Illumina MiSeq sequencing platform, generating *ca*. 2 million 75 bp paired-end reads. Due to the low abundance of endogenous DNA fragments observed, two-rounds of in-solution hybridization capture were performed to enrich mitochondrial DNA fragments^18,19^ using DNA baits generated from long-range PCR products of *Centrochelys sulcata* and *Chelonoidis chilensis* pooled at an equimolar rate. Details for long-range PCR, primer sequences, PCR conditions, and sequencing of enriched libraries are explained in the Supplementary Information.

### Processing of fresh material

DNA from fresh blood and tissue samples was extracted using commercial blood and tissue kits (Analytik Jena AG). DNA concentration and quality were assessed using a Qubit 3.0 Fluorometer (Thermo Fisher Scientific) and a 4200 TapeStation system (Agilent). For each sample, approximately 15 kbp were amplified in two overlapping long-range PCRs (for details see Supplementary Information). PCR products were sheared to approximately 150 bp with a Covaris M220 ultrasonicator, cleaned with the MinElute PCR Purification Kit (Qiagen), pooled at an equimolar rate, and built into single-indexed double-stranded Illumina sequencing libraries following Meyer and Kircher^20^. Sequencing was as described for old material and in the Supplementary Information.

### Mitogenome sequence assembly

Assembly of mitogenome sequences from the enriched and amplicon libraries involved adapter trimming with Skewer v0.2.2^21^, read merging (minimum length 35 bp), quality filtering (minimum Q-score 20), and duplicate removal with BBmap-suite 37.24 (https://sourceforge.net/projects/bbmap/)^22^. If necessary, subsampling with fastq-tools v 0.8 (https://github.com/dcjones/fastq-tools) was performed before the readpools were subjected to a two-step baiting and iterative mapping approach in MITObim^23^ with an allowed mismatch value of 2 and a reference sequence according to Table S4. Resulting scaffolds were visualised and checked for coverage (minimum per-site coverage: 3-fold) and assembly artefacts in Tablet^24^. Sequence length distribution of mapped reads was calculated with a customised awk command and Microsoft Excel. After assembly, PCR priming sites were removed from amplicon assemblies.

### Alignment and phylogenetic, divergence time, and biogeographic analyses

An automated preliminary alignment of our sequence data was performed using Clustal W 1.4^25^ as implemented in BioEdit 7.0.9.038, with default parameters^26^. This alignment was adjusted manually and sequences were annotated using MITOS^27^ and several published mitogenomes used for phylogenetic calculations (AF069423, DQ080042, DQ080048, FJ469674, KT613185, LT599485; Supplementary Information). Finally, each coding region was screened for internal stop codons using MEGA 7.0.21^28^, and problematic sequence features (e.g., stop codons, gene overlap, frameshifts) were removed (Supplementary Information).

Previously published mitogenomes of extant tortoise species were downloaded from GenBank and quality-checked. Unreliable or duplicate data were excluded from further analyses; reliable high-quality GenBank data for 10 taxa were included in our alignment to widen taxonomic coverage (Supplementary Information).

Our final alignment of 15,510 sites comprised 45 sequences corresponding to all extant genera of tortoises (Testudinidae), including representatives of all species groups of Testudininae, the clade to which *Cylindraspis* belongs^9,15^, and of all extant tortoise species from Madagascar and Aldabra. The two outgroup taxa represented the successive sister taxa of Testudinidae, Geoemydidae (*Mauremys reevesii*) and Emydidae (*Chrysemys picta*; Supplementary Information).

Phylogenetic relationships of the mitogenomes were examined with Maximum Likelihood (ML) and Bayesian Inference (BI) approaches using RAxML 8.0.0^29^ and MrBayes 3.2.6^30^. Divergence times were estimated using the uncorrelated lognormal relaxed clock models implemented in BEAST 1.8^31^ and constrained by four fossil calibrations. Ancestral ranges for tortoise lineages were inferred using the Maximum Likelihood framework implemented in BioGeoBEARS^32^, based on extant distribution ranges matching with 11 biogeographic areas and tailored dispersal models across time reflecting dispersal probabilities for six time intervals ranging from the early Eocene to the Plio-Pleistocene. Details are explained in the Supplementary Information.

## Results

Eight out of the 19 *Cylindraspis* samples produced high-quality data covering nearly the whole mitochondrial genome (15,335–15,348 bp length; Table S5). Only the control region and part of adjacent DNA coding for tRNAs were missing. The eight mitogenomes represented all five species of *Cylindraspis*. One mitogenome each was obtained for *C*. *peltastes*, *C*. *triserrata*, and *C*. *vosmaeri*, three for *C*. *indica* and two for *C*. *inepta*. Three additional samples (*C*. *inepta*, *C*. *peltastes*, *C*. *triserrata*) yielded complete cytochrome *b* sequences, whereas the data quality for eight further samples prevented their usage. All 16 fresh testudinid samples were processed successfully. Assembly details of individual samples and blanks are provided in the Supplementary Information. The aligned mitogenomes covered 15,510 sites.

**Figure 1:**
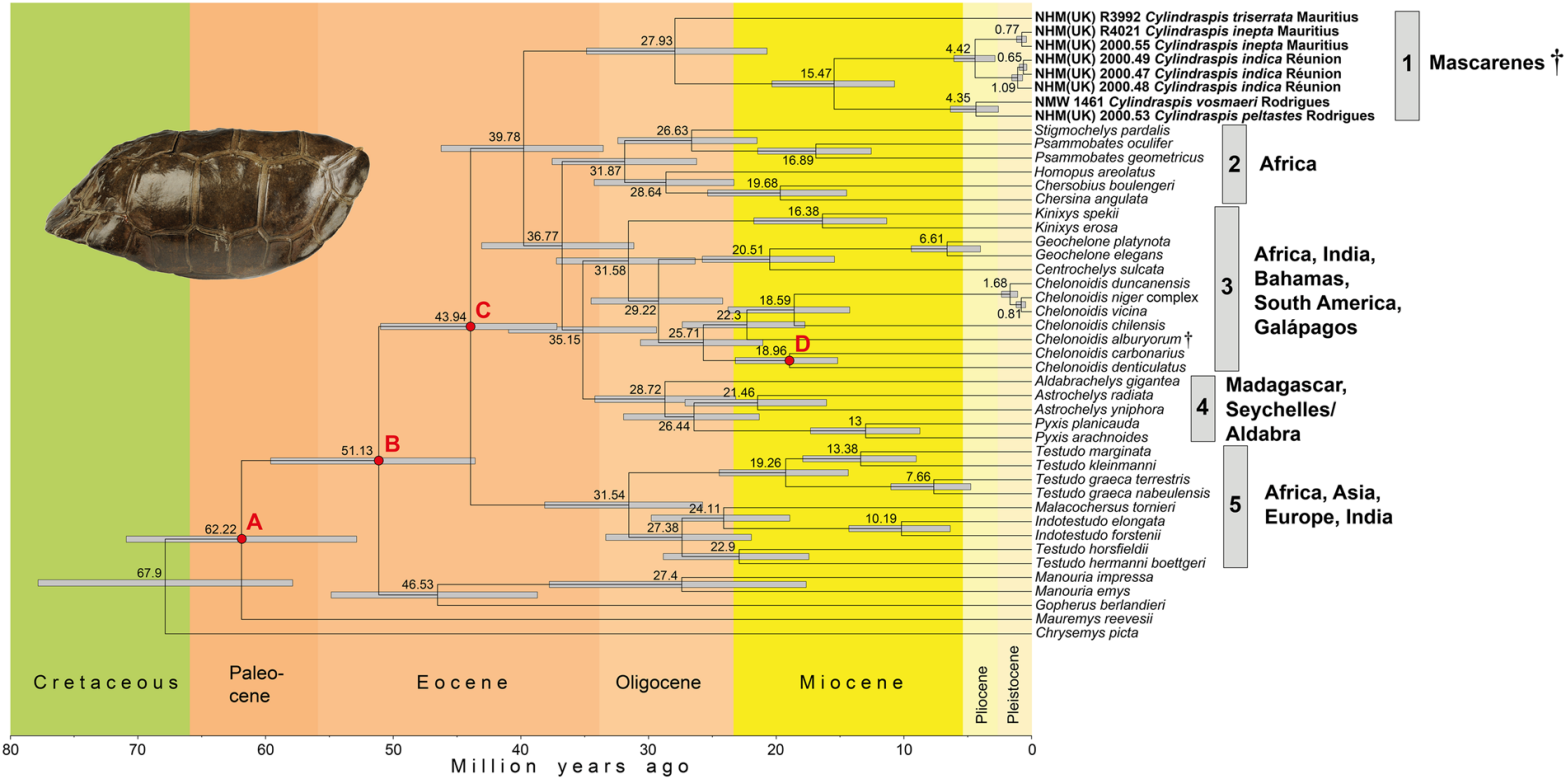
Fossil-calibrated time tree for *Cylindraspis* and all extant tortoise genera, rooted with *Chrysemys picta* based on 15,510 bp of the mitochondrial genome. *Cylindraspis* species in boldface with collection acronyms identifying individual specimens (NHM[UK] = Natural History Museum, London; NMW = Natural History Museum, Vienna). Dagger symbols denote extinct taxa. Geographic distributions of the five major testudinine clades (1–5) on the right. For nodes, inferred mean ages and 95% Highest Posterior Density intervals are shown. The red circles indicate fossil-based constraints: (A) *Hadrianus majusculus* Hay, 1904, 50.3–100.5 Ma; (B) *Cheirogaster maurini* Bergounioux, 1935 and *Gigantochersina ammon* (Andrews, 1904), 33.9–66.0 Ma; (C) *Cheirogaster maurini* Bergounioux, 1935, 33.9–47.8 Ma; (D) *Chelonoidis hesternus* (Auffenberg, 1971), 11.8–33.9 Ma. For details of calibration, see Supplementary Information. Inset: lateral view of the historic shell of *Cylindraspis vosmaeri* in the Natural History Museum, Vienna (NMW 1461); photo: Silke Schweiger.

The topologies of the ML and BI phylogenies were identical, with maximum support for most nodes. Most branching patterns conformed to expectations from previous studies using less sequence data^14,33,34^. However, the placement of *Gopherus* and *Manouria*, two genera often considered to represent together the sister group of all remaining tortoises^33,34^, was weakly resolved. These two genera were, with weak support, the successive sister taxa of the remaining genera, which constitute the clade Testudininae. In addition, branching patterns within the clade comprised of *Indotestudo*, *Malacochersus*, and *Testudo* were weakly resolved, except for the maximally supported *Testudo* sensu stricto clade. Here, *T*. *marginata* + *T*. *kleinmanni* together constituted the sister group of *T*. *graeca*, which was represented by two deeply divergent subspecies. The BEAST topology used for molecular dating and ancestral range analyses matched the ML and BI trees for all well-supported nodes but differed in the weakly resolved branching patterns in that *Gopherus* and *Manouria* were monophyletic. Moreover, *T*. *hermanni* and *T*. *horsfieldii*, and *Malacochersus* and *Indotestudo,* were sister taxa (Fig. 1 and Supplementary Information).

In all trees, *Cylindraspis* was monophyletic and sister to a diverse and deeply structured clade containing genera from sub-Saharan Africa (*Centrochelys*, *Chersina*, *Chersobius*, *Homopus*, *Kinixys*, *Psammobates*, *Stigmochelys*), India (*Geochelone*), Madagascar (*Astrochelys*, *Pyxis*), Aldabra (*Aldabrachelys*), and South America plus the Bahamas and Galápagos (*Chelonoidis*), i.e., the majority of the other Testudininae except for the clade containing *Malacochersus*, *Indotestudo* and *Testudo* that was sister to *Cylindraspis* and the remaining genera. Within *Cylindraspis*, *C*. *triserrata* from Mauritius was deeply divergent from the remaining four species (Fig. 1 and Supplementary Information). *Cylindraspis inepta*, also from Mauritius, was sister to *C*. *indica* from Réunion. Within the two and three mitogenomes of these two species occurred divergences resembling those observed among three mitogenomes of distinct species of Galápagos tortoises (*Chelonoidis duncanensis*, *C*. *niger* complex, *C*. *vicina*). The two *Cylindraspis* species from Rodrigues, *C*. *vosmaeri* and *C*. *peltastes*, were sister taxa with a genetic divergence similar to that between *C*. *indica* and *C*. *inepta*.

According to our fossil-calibrated molecular clock, *Cylindraspis* diverged from its sister group in the middle Eocene, with a mean estimate of 39.78 Ma and a 95% Highest Posterior Density (HPD) interval only slightly reaching into the lowermost Oligocene. The radiation of *Cylindraspis* was estimated to have commenced in the mid-Oligocene (divergence of *C*. *triserrata*; mean estimate 27.93 Ma). The divergence between the common ancestors of *C*. *inepta* and *C*. *indica* and of *C*. *vosmaeri* and *C*. *peltastes* was dated to a mean of 15.47 Ma (middle Miocene), whereas the two species pairs were inferred to have diverged in the early Pliocene (means of 4.42 Ma and 4.35 Ma, respectively). Our ancestral range analysis using BioGeoBEARS inferred Africa as the source region of *Cylindraspis* (Supplementary Information).

## Discussion

According to our analyses, the extinct genus *Cylindraspis* represents an ancient and deeply divergent clade of giant tortoises that separated from other tortoises in the Eocene (Fig. 1). There are no other extant tortoise taxa that are closely related to *Cylindraspis*, and this genus constitutes one of the five deeply divergent clades of testudinine tortoises. This phylogenetic placement categorically rules out the possibility of *Cylindraspis* having colonized the Mascarenes over the course of human history^5,9^.Our divergence dates for the primary lineages of Testudinidae (Fig. 1) match well with another recent estimate using mitogenomes but fewer taxa^14^, with most nodes of our study having slightly older mean values. According to our calculation including representatives of all extant testudinid genera and of all testudinine species groups, the majority of extant tortoise genera diverged in the Oligocene, with five major testudinine radiations in the Eocene and Oligocene corresponding to (1) the genus *Cylindraspis* from the Mascarenes; (2) genera from sub-Saharan Africa (*Chersina*, *Chersobius*, *Homopus*, *Psammobates*, *Stigmochelys*); (3) a diverse group including African genera (*Centrochelys*, *Kinixys*) and out of Africa dispersals to India (*Geochelone*), South America, the Bahamas and Galápagos (*Chelonoidis*); (4) a Western Indian Ocean radiation comprising taxa from Madagascar (*Astrochelys*, *Pyxis*) and Aldabra (*Aldabrachelys*); and (5) a group comprising genera from Mediterranean Africa, Europe and Asia (*Testudo*), sub-Saharan Africa (*Malacochersus*) and Asia plus India (*Indotestudo*). The former presence of tortoises of the clade Geochelona^15^ (i.e., the clade formed by 1–4) in the Eocene and Oligocene of Europe^15,35^ suggests that this group had a much wider distribution in the past, but a better understanding of the poorly documented Asian record of tortoises is needed to clarify if they were present in Asia or India as well as early as the late Paleogene.

**Figure 2:**
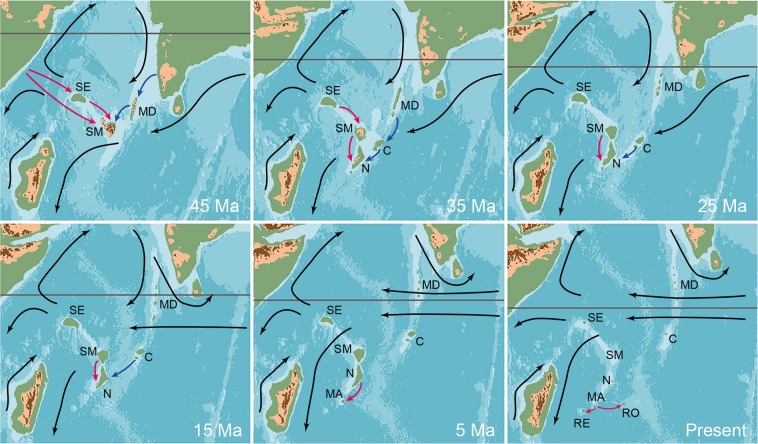
Paleogeographic reconstruction of the western Indian Ocean Basin highlighting changes from the late Eocene (45 Ma) to the present. The grey line connotes the approximate position of the equator; black arrows, prevalent surface currents; red and blue arrows, alternative colonization routes. Our model favours the red pathway, but the blue is more consistent with currents. It remains unclear whether the Seychelles Plateau was used as a stepping stone. Arrows are added to multiple time intervals to highlight ambiguity in regard to timing. The dispersal shown for the present took place much earlier. For Mauritius and Rodrigues multiple colonization events occurred. In addition to the persistent presence of anticlockwise gyres in the Mozambique Channel and the Indian Ocean, we posit the presence of an enclosed clockwise gyre in the Arabian Sea prior to tectonic movement of India north of the equator. In the Eocene sporadic surface current changes allowed overseas dispersal from northeast Mozambique and Tanzania to northern Madagascar^39^. Abbreviations: C – Chagos Plateau, MA – Mauritius, MD – Maldives Ridge, N – Nazareth Bank, RE – Réunion, RO – Rodrigues, SE – Seychelles Plateau, SM – Saya de Malha Bank. All maps were redrawn from Scotese^41^ using Photoshop CC 19.1.0 and then assembled and labelled using Illustrator 22.0.1.

Our BioGeoBEARS analyses reject Aldabra, Madagascar, the Seychelles Plateau or India as ancestral range and identify Africa as the source region of the Mascarene tortoises (Supplementary Information). Our time tree suggests that *Cylindraspis* diversified in the Oligocene, long before the Mascarene Islands had emerged from the ocean. However, the Réunion Hotspot formed a series of islands over the past 65 Ma^36,37^ (Fig. 2). The largest of these land masses (i.e., the Maldives Ridge, the Chagos Plateau, Saya de Malha Bank, and Nazareth Bank) are today only minute islands that are too small to support tortoise populations. Ocean drilling demonstrated that these islands are underlain by large plateaus consisting of subaerially formed basalts covered by reef limestone^36,37^. This indicates that these plateaus once were large islands that were flooded to become extensive atolls before sinking into the ocean (Fig. 2). We see two plausible routes of dispersal, one along the Réunion Hotspot island chain (Maldives, Chagos, Nazareth, Mascarenes), as in Mascarene stick insects^38^ and bolyeriid snakes^8^, the other aided by the Seychelles Plateau (Seychelles Plateau, Saya de Malha, Nazareth, Mascarenes). The former hypothesis appears to be more consistent with ocean currents but implies dispersal from India. The latter suggests dispersal from Africa, as supported by our model, but appears less consistent with currents. Moreover, the Seychelles Plateau as stepping stone received no support in our biogeographic analyses, implying that the ancestors of *Cylindraspis* became extinct or never occurred there. However, for the Eocene, the time period when *Cylindraspis* diverged, there is evidence for sporadic surface current changes allowing overseas dispersal from northeast Mozambique and Tanzania to northern Madagascar^39^. It is likely that this also supported the colonization of Saya de Malha and, perhaps, the Seychelles Plateau. In either case, dispersal occurred in relatively small steps within the expanding western Indian Ocean Basin and carried the *Cylindraspis* lineage to the Mascarene Islands via Nazareth. Our model differs from previous ones suggested for tortoises^9,10^ by embracing the paleogeography of the Western Indian Ocean.

In contrast to previous studies^8–10^, which suggested for *Cylindraspis* a Malagasy origin and diversification on Mauritius, we infer an African origin and radiation of up to three *Cylindraspis* lineages on Nazareth that later successfully colonized Mauritius once it emerged from the ocean floor. The evolution of several tortoise species appears likely on a dynamic island system as Nazareth, allowing for allopatry. We further hypothesize that the—at least for giant tortoises—high diversity of *Cylindraspis*, with two deeply divergent species each on relatively small islands (Mauritius and in particular Rodrigues), resulted from subsequent colonization events from Nazareth and within the Mascarenes, also involving ecological diversification of browsers (saddle-backed species) and grazers (domed species).

Our data show that the evolutionary divergence of *Cylindraspis* by far exceeded that of other genetically studied extant and extinct giant tortoise lineages from the Aldabra and Madagascar (*Aldabrachelys*) or the Bahamas and Galápagos (*Chelonoidis*; Fig. 1). Also the dodo (*Raphus cucullatus*) and the Rodrigues solitaire (*Pezophaps solitaria*), two other iconic species of the lost Mascarene fauna, were less divergent. These two sister taxa evolved only in the Miocene and were phylogenetically embedded in the Indo-Pacific clade of doves and pigeons with many extant survivors^40^. Thus, the extinction of *Cylindraspis* not only represents loss of one of the five major radiations of extant testudinine tortoises but also that of a truly ancient vertebrate lineage.

## Supplementary information

Supplementary Information
