# Supplementary material for: Ancient mitogenomics clarifies radiation of extinct Mascarene giant tortoises (Cylindraspis spp.)
Source: Sci Rep. 2019 Nov 25;9:17487. doi: 10.1038/s41598-019-54019-y (PMC6877638; doi:10.1038/s41598-019-54019-y)
Supplement: Supplementary file 1 — Supplementary Information [file 41598_2019_54019_MOESM1_ESM.pdf]

# Ancient mitogenomics clarifies radiation of extinct Mascarene giant tortoises (*Cylindraspis* spp.)

Christian Kehlmaier, Eva Graciá, Patrick D. Campbell, Margaretha D. Hofmeyr, Silke Schweiger, Albert Martínez-Silvestre, Walter Joyce, Uwe Fritz

*Scientific Reports*

## Supplementary Information

### Materials and Methods

Mitochondrial DNA data and quality check of GenBank/ENA data

All mitochondrial DNA sequences for testudinids longer than 13,000 bp available by 31 December 2018 were downloaded from GenBank/the European Nucleotide Archive (ENA), quality-checked and compared to our data. Among the downloaded data were sequences for *Aldabrachelys gigantea* (accession number KT613185), *Astrochelys yniphora* (JX317746), *Indotestudo elongata* (DQ656607), *Stigmochelys pardalis* (DQ080041), and three sequences tagged as 'unverified' for *Astrochelys radiata* (KJ489403), *Centrochelys sulcata* (KJ489404), and *Geochelone elegans* (KJ489405). These seven mentioned sequences were not included in our final calculations of near-complete mitogenomes, however. The *S. pardalis* mitogenome DQ080041 was shown to be of chimeric origin, containing a nuclear mitochondrial insertion (numt; Fritz *et al.* 2010), and has therefore been replaced for the present study by a newly generated sequence. The GenBank sequence KT613185 for *Aldabrachelys gigantea* has been produced by shotgun sequencing (Besnard *et al.* 2016), and we were concerned that it might also contain numt data. Therefore, this sequence has also been replaced by a new one generated for the present study. For *I. elongata* two GenBank sequences of high quality were available but we excluded DQ656607 and only used the other one (DQ080043) for calculations. Sequence JX317746, identified by Xiong *et al*. (2019) and in GenBank as *Astrochelys yniphora*, was excluded because it represents *A. radiata* instead of *A. yniphora*, and sequences KJ489403–489405 were excluded because of their poor quality. In the latter sequences, assembly issues were evident in all coding genes except atp8, ND4L and ND4. For instance, these three sequences had an identical 58-bp-long insertion in the cytochrome *b* (cyt *b*) gene which is presumably of bacterial origin (Fig. S1). In addition, there are large deletions (KJ489403: 528 bp; KJ489404: 514 bp; KJ489405: 511 bp) present at or near the start of COI (Fig. S2).

Thus, our final alignment of near-complete mitochondrial genomes comprised 45 sequences corresponding to 42 taxa. Twenty-four of the 45 mitogenomes were generated for the present study, including eight data sets for the five extinct *Cylindraspis* species. *Cylindraspis indica* was represented by three and *C. inepta* by two near-complete mitogenomes, the remaining three species by one each. *Chrysemys picta* (AF069423, used for tree rooting) and *Mauremys reevesii* (FJ469674) served as outgroup taxa. *Mauremys reevesii* is a member

of the family Geoemydidae and *C. picta* of the Emydidae, which are successive sister taxa of the Testudinidae (Shaffer *et al.* 2017). The ingroup contained representatives of all extant genera of the Testudinidae family, including all extant taxa from Madagascar and Aldabra and representatives of all species groups within the Testudininae subfamily to which *Cylindraspis* belongs (Le & Raxworthy 2017; Vlachos & Rabi 2018):

*Aldabrachelys gigantea** – LR697067; MTD 18707
*Astrochelys radiata** – LR697068; MTD 18660
*Astrochelys yniphora** – LR697069; MTD 15998
*Centrochelys sulcata* – LT599487
*Chelonoidis alburyorum* – LT599482
*Chelonoidis carbonarius* – LT599483
*Chelonoidis chilensis* – LT599484
*Chelonoidis denticulatus* – LT599485
*Chelonoidis duncanensis* – MG912820
*Chelonoidis niger* complex – JN999704
*Chelonoidis vicina* – LT599486
*Chersina angulata** – LR697070; MTD 13772
*Chersobius boulengeri** – LR697071; MTD 15558
*Cylindraspis indica** – LR697059; NHM(UK) 2000.47
*Cylindraspis indica** – LR697060; NHM(UK) 2000.48
*Cylindraspis indica** – LR697061; NHM(UK) 2000.49
*Cylindraspis inepta** – LR697062; NHM(UK) R4021
*Cylindraspis inepta** – LR697063; NHM(UK) 2000.55
*Cylindraspis peltastes** – LR697064; NHM(UK) 2000.53
*Cylindraspis triserrata** – LR697065; NHM(UK) R3992
*Cylindraspis vosmaeri** – LR697066; NMW 1461
*Geochelone elegans** – LR697072; MTD 6057
*Geochelone platynota** – LR697073; MTD 4059
*Gopherus berlandieri** – LR697074; MTD 17171
*Homopus areolatus** – LR697075; MTD 15479
*Indotestudo elongata* – DQ080043
*Indotestudo forstenii* – DQ080044
*Kinixys erosa** – LR697076; MTD 15816
*Kinixys spekii** – LR697077; MTD 17037
*Malacochersus tornieri* – DQ080042
*Manouria emys* – DQ080040
*Manouria impressa* – EF661586
*Psammobates geometricus** – LR697078; MTD 13895
*Psammobates oculifer** – LR697079; MTD 18196
*Pyxis arachnoides** – LR697080; MTD 18661
*Pyxis planicauda** – LR697081; MTD 1244
*Stigmochelys pardalis** – LR697082; MTD 16076
*Testudo graeca nabeulensis* – DQ080049
*Testudo graeca terrestris* – DQ080050
*Testudo hermanni boettgeri* – DQ080046
*Testudo horsfieldii* – DQ080045
*Testudo kleinmanni* – DQ080048
*Testudo marginata* – DQ080047

Taxon names are followed in this list by GenBank/ENA accession numbers. Samples processed for this study bear asterisks. For these samples, lab codes (MTD) or numbers of museum vouchers follow accession numbers.

In addition, a 1,143-bp-long alignment was examined, containing 25 complete and 27 partial sequences of the cyt *b* gene. For this phylogenetically informative gene, three additional *Cylindraspis* sequences were obtained from our material. The alignment contained as outgroup *Gopherus berlandieri* (LR697074), and the ingroup consisted of all available cyt *b* sequences for *Cylindraspis* specimens from the present study and Austin & Arnold (2001).

Sequences of representatives of all extant testudinid taxa from Madagascar and Aldabra plus a 405-bp-long sequence of the extinct Malagasy species *Aldabrachelys grandidieri* from Austin *et al.* (2003) were included. For comparative purposes, representatives of two other island radiations of extant and extinct giant tortoises were added (*Aldabrachelys* from Madagascar, extinct, and Aldabra; *Chelonoidis* spp. from the Bahamas, extinct, and Galápagos plus their extant continental South American congeners):

*Aldabrachelys gigantea* – AF371241, AF371242, KT613185, LR697067*
*Aldabrachelys grandidieri* – AF371240
*Astrochelys radiata* – AF020897, AF371239, LR697068*
*Astrochelys yniphora* – AF020896, LR697069*
*Chelonoidis abingdonii* – AF192932
*Chelonoidis alburyorum* – LT599482
*Chelonoidis becki* – JN637211
*Chelonoidis carbonarius* – LT599483
*Chelonoidis chilensis* – LT599484
*Chelonoidis chathamensis* – AF192931
*Chelonoidis darwini* – AF192940
*Chelonoidis denticulatus* – LT599485
*Chelonoidis donfaustoi* – AY097816
*Chelonoidis duncanensis* – MG912820
*Chelonoidis ephippium* – JN637180
*Chelonoidis hoodensis* – AF192933
*Chelonoidis microphyes* – AF192938
*Chelonoidis niger* complex – JN637231, JN999704
*Chelonoidis phantasticus* – JN637228
*Chelonoidis porteri* – JN637214
*Chelonoidis vicina* – LT599486
*Cylindraspis indica* – AF371243, AF371244, LR697059–LR697061*
*Cylindraspis inepta* – LR694548*, LR697062*, LR697063*
*Cylindraspis peltastes* – AF371253, AF371254, LR694549*, LR697064*
*Cylindraspis triserrata* – AF371248, LR694550*, LR697065*
*Cylindraspis vosmaeri* – AF371257, AF371259, AF371260, LR697066*
*Pyxis arachnoides* – AF020894, LR697080*
*Pyxis planicauda* – AF020895, LR697081*

For individual lengths and voucher numbers of the *Cylindraspis* sequences, see Table S5. Accession numbers with asterisks indicate sequences produced for the present study.

## Amplicon sequencing

For fresh samples with high molecular weight DNA, amplicon sequencing of the mitochondrial genome was conducted. For each sample, two long-range PCR reactions were performed (LR1 and LR2) yielding amplicons with an overlap of at least 106 bp and an individual length of approximately 7,100–10,450 bp, depending on the primer combination. For each long-range PCR, a 50 µl volume was used, containing 3.6–32.0 ng of DNA and 1 unit of *TaKaRa LA* Taq DNA Polymerase, Hot-Start Version (Clontech Laboratories Inc., Mountain View, CA, USA), and the reaction mixture recommended by the manufacturer. PCR conditions comprised initial denaturation at 93°C for 3 min, followed by 30–40 cycles of 93°C for 20 sec, 50–55°C for 30 sec, 68°C for 12 min, and a final elongation step at 68°C for 20 min. For primer sequences, amount of DNA template, number of repetitive PCR cycles, annealing temperatures, and fragment lengths see Tables S6 and S7. PCR products were visualised and, if necessary, excised from a 2% agarose gel and purified using the NucleoSpin Gel and PCR Clean-up Kit (Macherey-Nagel GmbH & Co. KG, Düren, Germany). The combined long-range

PCR products covered most of the mitochondrial genome from tRNA-Phe (situated before 12S) to tRNA-Thr (situated after cyt *b*), missing out tRNA-Pro and the control region.

The authenticity of the long-range PCR products was verified by Sanger-sequencing part of the 12S and cyt *b* genes with well-established internal primers (Table S6) following standard procedures (Fritz *et al*. 2014). For cycle sequencing, the total reaction volume of 10 µl contained 2 µl sequencing buffer, 1 µl premix, 0.5 µM of the respective primer, 1 µl DNA template, and ultrapure $H_2O$. Using the ABI Prism Big Dye Terminator v.3.1 Cycle Sequencing Kit (Applied Biosystems, Foster City, CA, USA), 25 cycles were performed at 96°C for 10 sec, 50°C for 5 sec and 60°C for 4 min. Reaction products were purified by gel filtration using the Performa DTR V3 96-Well Short Plate Kit (EdgeBio, Gaithersburg, MD, USA) and 400 µl of a 5% Sephadex solution (GE Healthcare, Munich, Germany). Sequencing was performed in-house on an ABI 3730 Genetic Analyser (Applied Biosystems).

## Authenticity of mtDNA sequenced for the present study

For fresh samples, long-range PCRs and subsequent amplicon sequencing were conducted to minimize the risk of sequencing nuclear copies of mitochondrial DNA (numts; Bensasson *et al.* 2001; Fritz *et al*. 2012; Cui *et al*. 2013). During mitogenome assembly of *Cylindraspis* and fresh samples, a strict mismatch threshold of 2 was selected to prevent the integration of divergent reads of possible nuclear origin. Base frequencies of all sequences obtained for the present study, as well as their ratios of synonymous and non-synonymous substitutions, corresponded to expectations for mtDNA. In addition, protein-coding genes contained no internal stop codons, and nucleotides successfully translated into amino acids. Therefore, we are confident to have sequenced authentic mtDNA and not numts.

## Annotation of the alignment of mitogenomes (45 sequences)

To facilitate partitioning (Table S8), a somewhat shorter alignment was used for phylogenetic analyses: (1) stop codons of coding genes were excluded as these do not code for any amino acid; (2) gene overlap was deleted in seven instances because these short regions could not be identified with a single gene and may have evolved differently; where necessary, adjacent codon positions also had to be deleted in order to maintain an intact reading frame; (3) alignment positions that cause frameshifts in coding regions were removed (once in ND1, twice in COI, once in ND3, once in ND4, and once in ND6); and (4) 23 intergenic spacer regions were excluded. Those regions ranged from 1–16 bp, with the exception of a 159-bp-long indel between ND5 and ND6 present only in the outgroup.

In addition, GenBank sequence JN999704 was slightly modified before usage. This partial mitogenome was assembled by 454 pyrosequencing (Lourenço *et al.* 2011) and contains several stretches of unknown positions (stretches of N), which were not recovered by PCR-based Sanger sequencing, as was the case for other samples in the original publication. Three issues emerged which were considered by us to be nothing more than sequencing/assembling artefacts, which consequently have been altered manually for calculations:

(1) At the beginning of COI, the sequence is one triplet too short, without producing any internal stop codons. However, in accordance to the general picture of the alignment, this triplet can be divided into three individual indels, which were filled up with Ns (Fig. S3).
(2) There is a 37-bp-long deletion in the DNA coding for tRNA-Arg, which was filled with Ns, as the specimen would lack this tRNA otherwise.
(3) At the end of cyt *b*, the sequence had a unique internal stop codon (4th last codon), which was amended to match the common pattern (Fig. S4).

## Phylogenetic analyses

Phylogenetic relationships of the mitogenomes were inferred using RAxML 8.0.0 (Stamatakis 2014) and MrBayes 3.2.6 (Ronquist *el al*. 2012). The best evolutionary models (Table S9) and partitioning schemes were determined with PartitionFinder2 (Lanfear *et al.* 2016) and the Bayesian Information Criterion. Three different partition schemes were examined: (1) unpartitioned—1 partition; (2) gene-partitioned, i.e., 13 coding genes, 12S, 16S, and tRNAs combined—16 partitions; and (3) codon-partitioned, i.e., 13 times 3 codon positions extra, 12S, 16S, and tRNAs combined—42 partitions. For Maximum Likelihood (ML) and Bayesian Inference (BI), the codon-partitioned data set was selected. For ML, five independent searches were carried out using the GTR+G substitution model, different starting conditions, and the rapid bootstrap option. Subsequently, 1000 non-parametric thorough bootstrap replicates were calculated and the values plotted against the best tree. For BI, two parallel runs (each with four chains) were performed with 10 million generations (burn-in 0.25; print frequency 1000; sample frequency 500). Calculation parameters were analysed using Tracer 1.7.1 (Rambaut *et al.* 2018). The cyt *b* alignment was explored only with RAxML for a codon-partitioned alignment, as suggested by PartitionFinder2, using the GTR+G model. As alternatives, an unpartitioned and a 'third-codon-position-extra' scheme were tested. In addition, uncorrected *p* distances were calculated in MEGA7 (Kumar *et al.* 2016) using the pairwise deletion option.

## Divergence time analyses

Molecular dating relied on the uncorrelated lognormal relaxed clock models implemented in BEAST 1.8 (Drummond *et al.* 2012) using a Yule tree with the HKY substitution model and four rate categories. MCMC chains ran for 20 million generations, with parameters and trees sampled every 20,000 generations. Tracer 1.6 (Rambaut & Drummond 2007) served to check for convergence of the runs using Effective Sample Sizes (ESS) of parameters, resulting in ESSs over 200 after discarding 10% of the initial trees as burn-in. Trees were summarized using TreeAnnotator 1.8 and the maximum clade credibility tree and mean node height options.

Four nodes were calibrated using priors under lognormal distributions (Table S10). Following Joyce *et al.* (2013), the total clade of Testudinidae (= crown Testuguria) was constrained with a minimum at 50.3 Ma (i.e., the top of the Wasatchian North American Land

Mammal Age) and a maximum at 100.5 Ma (i.e., the base of the Late Cretaceous). This constraint was based on the occurrence of the unambiguous pan-testudinid *Hadrianus majusculus* Hay, 1904 in sediments referred to the Wasatchian North American Land Mammal Age. The phylogenetic position of *H. majusculus* as a stem testudinid, not a crown testudinid, was recently confirmed (Vlachos & Rabi 2018). Following Kehlmaier *et al.* (2017) the node formed by the extant *Chelonoidis carbonarius* and *C. denticulatus* was calibrated based on the fossil *C. hesternus* (Auffenberg, 1971), which was collected from sediments referred to the Laventan South American Land Mammal Age. However, we establish the minimum at 11.8 Ma, not 12.55 Ma, based on the minimum published age for the Laventan, and implemented a maximum of 33.9 Ma corresponding to the base of the Oligocene, as no tortoises have been reported from South America prior to the Oligocene (de la Fuente *et al.* 2018).

The minimum age of crown Testudinidae was constrained at the top of the Eocene (33.9 Ma) using the late Eocene (Priabonian) *Cheirogaster maurini* Bergounioux, 1935 and *Gigantochersina ammon* (Andrews, 1904), which were recently hypothesized to be nested deeply within Testudinidae (i.e., crown Geochelona) by Vlachos & Rabi (2018). Although the presence of a supracaudal scute (i.e., fused marginal scutes XII) is not necessarily a synapomorphy of this clade (see Crumly 1985 versus Vlachos & Rabi 2018), it is notable that this derived character unique to tortoises has a broad presence in Europe by the end of the Eocene, which further supports the assertion that crown Testudinidae was established by then. As the maximum for this clade, the base of the Tertiary (66.0 Ma) was used because no "tortoise," stem or crown, has as of yet been reported from the Mesozoic.

Finally, the minimum age of crown Testudininae (i.e., the clade formed by all extant tortoises to the exclusion of *Manouria* and *Gopherus*) was constrained by reference to the late Eocene (Priabonian) *C. maurini*, which, as noted above, was recently hypothesized to be nested within the clade Geochelona (Vlachos & Rabi 2018). In contrast to other, potential, geochelonans from the late Eocene, *C. maurini* already exhibits the absence of a cervical scute, a character that is uniquely found in geochelonans among cryptodires. As no tortoises with derived characters have been reported from prior to the late Eocene, the maximum for this clade was established at 47.8 Ma.

## Biogeographic analyses

Ancestral ranges were inferred using the Maximum Likelihood framework implemented in the R package BioGeoBEARS (Matzke 2013) and run in RASP 4 (Yu *et al.* 2015). BioGeoBEARS allows for estimating ancestral ranges of taxa using and comparing alternative models: The LAGRANGE dispersal–extinction–cladogenesis (DEC) model, a likelihood version of dispersal-vicariance model (DIVALIKE) and a likelihood version of the BAYAREA model (BAYAREALIKE). These models make different assumptions about anagenetic and cladogenetic processes impacting the results (Matzke 2014). The best-fit model was selected based on Akaike Information Criterion scores, resulting in the selection of the DIVALIKE model. Statistics from BioGeoBEARS analysis for the three models are shown in Table S11.

For BioGeoBEARS, the time-calibrated BEAST tree and a matrix of extant geographic distributions in presence-absence format corresponding to the following 11 biogeographic areas were used to follow the geological boundary of extant continents and islands, not recent biogeographic realms: (A) Asia, without India; (B) Europe; (C) India; (D) Africa, including Arabia; (E) Madagascar; (F) North America; (G) South America; (H) Galápagos; (I) Caribbean; (J) Mascarenes; and (K) Aldabra + Seychelles. Paleogeographic differences were acknowledged using six different time bins: (i) early Eocene (> 47.8 Ma); (ii) middle + late Eocene (33.9–47.8 Ma); (iii) Oligocene (23.0–33.9 Ma); (iv) early Miocene (16.0–23.0 Ma); (v) middle + late Miocene (5.3–16.0 Ma); and (vi) Plio-Pleistocene (< 5.3 Ma). Dispersal probabilities between areas were scaled from 0 (e.g., for the dispersal to areas that were not yet formed) to 1 (connected land masses). A weighted probability matrix was established to assess the likelihood of dispersal between areas: 1 (land masses connected), 0.75 (land masses poorly connected by land or separated by minor oceanic barriers equivalent to the distance of the Galápagos Islands from South America or of Aldabra to Madagascar), 0.5 (land masses separated by large terrestrial barriers or by intermediate oceanic barriers along the currents), 0.1 (land masses not connected by land, but separated by extensive oceanic barriers along the currents), 0.01 (land masses not connected by land or direct oceanic currents), and 0 (dispersal impossible, as one of the two land masses does not exist). The values for each time bin were then scored by explicit reference to global paleogeographic reconstructions (Scotese 2013) in combination with the ages of various islands and plateaus found throughout the Western Indian Ocean (Duncan *et al*. 1989). The data matrix is available through Dryad https://doi.org/10.5061/dryad.08kprr4xz.

## Results and Discussion

### NGS data for *Cylindraspis*

Compared to shotgun sequencing, hybridization capture increased the content of endogenous mtDNA for the *Cylindraspis* sample NHM(UK) 2000.49 by two orders of magnitude, i.e., from 0.02% (readpool: 1.72 million reads) to 2.83% (readpool: 1.89 million reads; Table S12). Using hybridization capture, the historic tissue sample and seven subfossil bone samples of *Cylindraspis* produced mitochondrial DNA data of high quality that allowed assembling near-complete mitogenomes. The remaining eleven bone samples yielded patchy contig-sequences with considerably lower coverage and many ambiguous sites. Yet, cyt *b* data of four additional bone samples were of sufficient quality to be examined. These cyt *b* sequences were aligned with the cyt *b* data of the eight *Cylindraspis* mitogenomes, with stop codons excluded, resulting in a length of 1,143 bp. This allowed us to compare our data for these *Cylindraspis* samples with cyt *b* data (405 bp) for 11 specimens from Austin & Arnold (2001) produced by Sanger sequencing (Table S1). For 10 specimens (NHM[UK] 2000.47–2000.49, 2000.52–2000.55, R3992, R4021, NMW 1461), our sequences were identical with those from Austin & Arnold (2001). However, for the specimen (NHM[UK] 2000.51) with the lowest sequence quality, 15 differences were observed. Therefore, our sequence was discarded and the GenBank sequence AF371254 from Austin & Arnold (2001) used for further

analyses. For another sample morphologically identified as *C. inepta* (NHM[UK] R3991), Austin & Arnold (2001) could not generate a cyt *b* sequence. Our NGS approach yielded the complete cyt *b* gene for this tortoise and unambiguously identified it as a *C. triserrata*.

## Phylogeny of Testudininae based on mitogenomes

Our phylogenetic analyses revealed five deeply divergent clades within Testudininae, one of which corresponded to *Cylindraspis* (Figs 1 and S5). A well-supported clade comprised of *Indotestudo* (Asia, India), *Malacochersus* (Africa), and *Testudo* (Africa, Europe, Asia) constitutes the sister taxon of the remaining four deeply divergent clades. The clade corresponding to *Cylindraspis* is sister to the remaining three deeply divergent clades. The successive sister is a clade consisting of the African genera *Chersina*, *Chersobius*, *Homopus*, *Psammobates*, and *Stigmochelys*. This clade is sister to the crown group formed by another two deeply divergent clades. One contains *Centrochelys* (Africa), *Chelonoidis* (South America, Galápagos, extinct: Caribbean), *Geochelone* (India), and *Kinixys* (Africa). The other clade comprises taxa from the Western Indian Ocean (Aldabra, Madagascar), i.e., *Aldabrachelys*, *Astrochelys*, and *Pyxis*.

The branching patterns within the clade containing *Indotestudo*, *Malacochersus*, and *Testudo* are not well resolved, with weak support for some nodes. *Testudo* is not monophyletic, as in a previous analysis of mitogenomes (Parham *et al*. 2006). However, two other studies using mitochondrial and nuclear DNA sequences (Le *et al*. 2006; Fritz & Bininda-Emonds 2007) and another one using mitogenomes (Kehlmaier *et al*. 2017) found *Testudo* monophyletic, albeit with weak support. It is obvious that resolving this contradictory pattern is beyond the scope of the present study and that further investigations are needed.

## A cyt *b* phylogeny of giant tortoises

Our cyt *b* data set included all available 19 sequences for *Cylindraspis* from the present study and Austin & Arnold (2001), i.e., three sequences for *C. inepta*, five for *C. indica*, four for *C. peltastes*, three for *C. triserrata*, and four for *C. vosmaeri*. The alignment included also sequences for all extant tortoise species from the Western Indian Ocean (*Aldabrachelys gigantea*, *Astrochelys radiata*, *A. yniphora*, *Pyxis arachnoides*, *P. planicauda*), the extinct *Aldabrachelys grandidieri* from Madagascar, and for another genus (*Chelonoidis*) with extant and extinct giant tortoise species from the Bahamas (*C. alburyorum*, extinct) and Galápagos (*C. niger* complex) plus their continental South American congeners *C. carbonarius*, *C. denticulatus*, and *C. chilensis*. *Gopherus berlandieri* served for tree rooting. In comparison to the trees derived from the mitogenomes (Fig. S5), some nodes of the ML tree based on cyt *b* alone (Fig. S6) were only weakly supported, and the branching pattern for the tortoise taxa from the Western Indian Ocean (Madagascar, Aldabra) was only weakly resolved. However, the monophyly of *Cylindraspis* received reasonable bootstrap support of 76. Le & Raxworthy (2017) doubted the authenticity of the sequences of *C. triserrata* produced by Austin & Arnold (2001) because they clustered in their re-analyses with *Chelonoidis*. However, the results of our phylogenetic analyses and the comparison of the sequences with ours provide unambiguous evidence that the short cyt *b* sequences produced by Austin & Arnold (2001)

are authentic and that *C. triserrata* is a deeply divergent species of *Cylindraspis* (see also main text). We abstain from speculations about the results reported in Le & Raxworthy (2017). Within *Cylindraspis*, the placement of our 1,143-bp-long cyt *b* sequence of the historic specimen of *C. vosmaeri* (NMW 1461) clearly differed from that of three sequences from Austin & Arnold (2001), rendering *C. vosmaeri* paraphyletic with respect to *C. peltastes*. Unfortunately, the three other specimens of *C. vosmaeri* used by Austin & Arnold (2001) were either not available for study or yielded no results. The deep divergence of the near-complete mitogenomes of *C. peltastes* and *C. vosmaeri* (Fig. S5) suggests that missing information, due to the short lengths (less than 50%) of the sequences from Austin & Arnold (2001) compared to our cyt *b* sequence of *C. vosmaeri*, is responsible for the paraphyly of *C. vosmaeri*. Nevertheless, this situation warrants further research involving broader sampling, especially because the occurrence of two distinct giant tortoise species (*C. peltastes*, *C. vosmaeri*) on a small island like Rodrigues is unexpected and unique in comparison to other fossil and extant giant tortoises.

Compared to *Cylindraspis*, the sequence divergences between giant tortoise species from Galápagos (*Chelonoidis niger* complex) are shallow and also distinctly lower than the divergence between two subspecies of *Testudo graeca* (Figs S5–S8). The divergences among Galápagos tortoises resemble those observed within individual *Cylindraspis* species, supporting recent doubts to whether the species status is justified for the distinct giant tortoise populations from Galápagos or not (Loire & Galtier 2017; Galtier 2019).

## Colonization history

The BioGeoBEARS analysis selected the DIVALIKE model as best supported (Table S11). The resulting ancestral area estimation (Fig. S9) revealed Africa as the ancestral range of *Cylindraspis* (node 75) and clearly discarded Aldabra, Madagascar, the Seychelles Plateau or India as source regions (ML probability of ancestral ranges: Mascarene-Africa = 0.88; Africa = 0.1). The extant and extinct tortoises from Madagascar, the granitic Seychelles, and Aldabra represent another clade (node 60), suggesting that these land masses were not stepping stones for the dispersal process of *Cylindraspis* because then subsequent extinction and replacement would have to be postulated.

The place of origin for the Testudinidae (node 87) remained unresolved (Fig. S9). This is not surprising, as the testudinid lineages have a broad distribution across the Northern and Southern Hemispheres. However, the basal tortoise lineages were widely distributed only over the Northern Hemisphere (Vlachos 2018; Vlachos & Rabi 2018). Given the preponderance of basal testudinoids in the Late Cretaceous to the Paleocene of Asia, independent paleontological evidence suggests an Asian origin for the clade (e.g., Sukhanov 2000) with dispersal in the Eocene to Africa, Europe, and North America (e.g., Joyce *et al*. 2016) and in the Oligocene from Africa to South America (this study; see also Kehlmaier *et al*. 2017). On the other hand, Africa was clearly suggested as the ancestral region for the clade Testudininae (node 84).

The biogeographic history for the clade comprising *Indotestudo*, *Malacochersus*, and *Testudo* (= clade Testudona of Parham *et al*. 2006), corresponding to node 83, was not well

resolved, even though Europe and Asia were inferred as most likely source regions. This is not surprising, as the clade mostly developed in the Neogene when these land masses had fully merged to form a single functional continent. The fossil record of the group is mostly restricted to these land masses as well (e.g., Sukhanov 2000; de Lapparent de Broin 2001; Danilov 2005; Brinkman *et al*. 2008) but is in dire need of revision.

The clade that opposes Testudona was recently named Geochelona by Vlachos & Rabi (2018), but the phylogenetic definition was worded in such a way that *Cylindraspis* is excluded. As these turtles can be considered recent, because they only went extinct during historic times, we here slightly modify the definition of Geochelona to refer to the most inclusive crown clade of tortoises that includes *Geochelone elegans* (Schoepff, 1795), but not *Testudo graeca* Linnaeus, 1758, corresponding to node 75 in Figure S9.

Africa and the Mascarenes were inferred by the analysis as possible ancestral areas for Geochelona, but this must be an artefact of the method, as the two land masses were never connected. Geochelona therefore likely originated in Africa. From there, the clade colonized India, Madagascar with Aldabra and the granitic Seychelles, South America with the Caribbean and the Galápagos Islands, and the Mascarenes. Except for the Mascarenes, this is in broad accordance with biogeographic models developed over the course of the last twenty years based on molecular (e.g., Le *et al*. 2006) or fossil (e.g., Joyce *et al*. 2016) evidence.

The land mass "Mascarenes" implemented in our biogeographic analysis includes all islands that were formed by the Réunion Hotspot during the last 65 Ma. The unambiguous result that *Cylindraspis* originated on this land mass in the Oligocene is therefore not contradicted per se by the young age of the current Mascarene Islands. The basal divergence of *Cylindraspis* giving rise to the *C. triserrata* lineage occurred approximately 28 Ma ago (Fig. 1), which postdates the formation of the Nazareth Plateau by about 8 Ma. As this is the land mass closest to the extant Mascarenes, the radiation of the group is likely to have commenced there. The lineages of *C. inepta* + *C. indica* and *C. vosmaeri* + *C. peltastes* diverged approximately 15.5 Ma ago (Fig. 1), which still precedes the formation of Mauritius by 7 Ma (all ages used herein taken from Duncan *et al*. 1989 and Duncan & Hargraves 1990). We therefore suggest that this divergence occurred on Nazareth as well. Thus, at least three lineages dispersed independently and subsequently from Nazareth to the Mascarenes. The oldest island, Mauritius, emerged from the sea floor about 8 Ma ago and it seems likely that it served as a stepping stone for the colonization of the much younger islands of Réunion and Rodrigues. Both are thought to have formed over the course of the last 2 million years, but the marine ridges that underlie the present-day islands are often much older. It cannot be excluded that additional small land masses were exposed on which the individual species originated. We believe that the evolution of five distinct giant tortoise species on the Mascarenes reflects a complicated interplay of multiple overseas dispersals from Nazareth to a dynamic island system in the region of the present-day Mascarenes and within the Mascarenes. The three oldest lineages, i.e., *C. triserrata* and the ancestral lineages of *C. inepta* + *C. indica* and *C. vosmaeri* + *C. peltastes* most likely arrived already from Nazareth, while the

divergence of the latter two species pairs is thought to reflect dispersal and subsequent vicariance within the Mascarene island system.

It remains unclear whether Saya de Malha and Nazareth were reached by the ancestor of *Cylindraspis* via the Seychelles Plateau or directly from Africa (Fig. 2). Today Aldabra is inhabited by another genus of giant tortoises (*Aldabrachelys*), which once occurred also on Madagascar and the granitic Seychelles (TEWG 2015; TTWG 2017), suggesting either that the Seychelles Plateau was circumvented during the colonization process or, less likely, that *Cylindraspis* became later extinct there.

Austin & Arnold (2001) discussed and disregarded the possibility that *Cylindraspis* dispersed to the Mascarenes from Southeast Asia across the entire Indian Ocean. Overseas dispersal from Southeast Asia has been considered for the Round Island boas (Bolyeriidae; Hawlitschek *et al*. 2017), and an origin in the Eastern Indian Ocean has been inferred for the ancestors of Mascarene stick insects (Bradler *et al*. 2015) and some Mascarene lizards (*Nactus*, *Leiolopisma*; Austin & Arnold 2006; Arnold & Bour 2008). An origin in Southeast Asia is made plausible for *Cylindraspis* by the presence of the giant fossil tortoises of the genus *Megalochelys* in the Plio-Pleistocene of Indonesia, the Philippines, and continental South and Southeast Asia (TEWG 2015). *Megalochelys* is characterized by strongly forked gulars (Setiyabudi 2009), a character also known to occur in *Cylindraspis triserrata* but not in the remaining *Cylindraspis* species (Gadow 1894; Bour *et al*. 2014). However, the firm placement of *Megalochelys* as sister to *Centrochelys sulcata* based on morphological evidence (Vlachos & Rabi 2018) implies that this feature evolved homoplastically in these two lineages. The sister group relationship of *Centrochelys* and *Megalochelys* also rules out the argument that the latter is related to the ancestor of *Cylindraspis* because then a sister group relationship of *Cylindraspis* and *Megalochelys* would be expected. However, instead the two genera belong to two deeply divergent testudinine clades (clade 1 and 3 in Fig. 1).

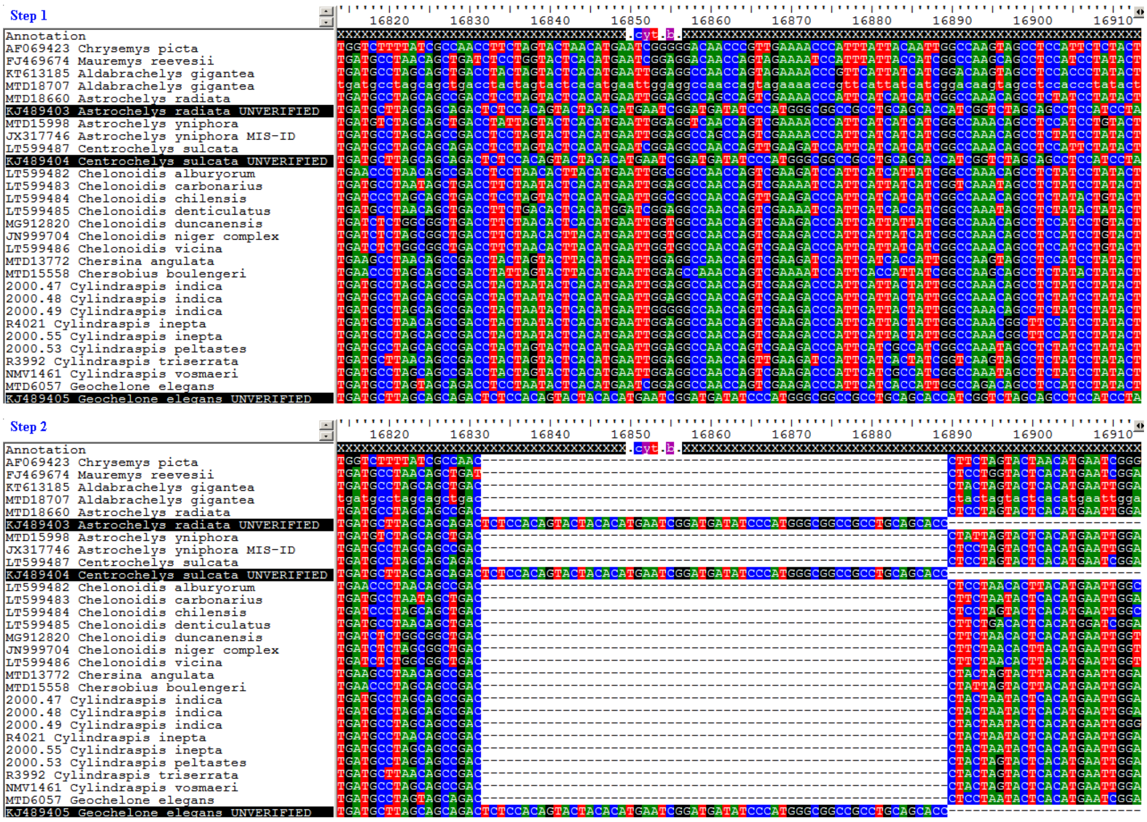


**Figure S1.** Example for an assembly artefact in KJ489403–KJ489405. Shown is a 58-bp-long insertion within the cyt *b* gene identical for all three sequences and of presumable bacterial origin.

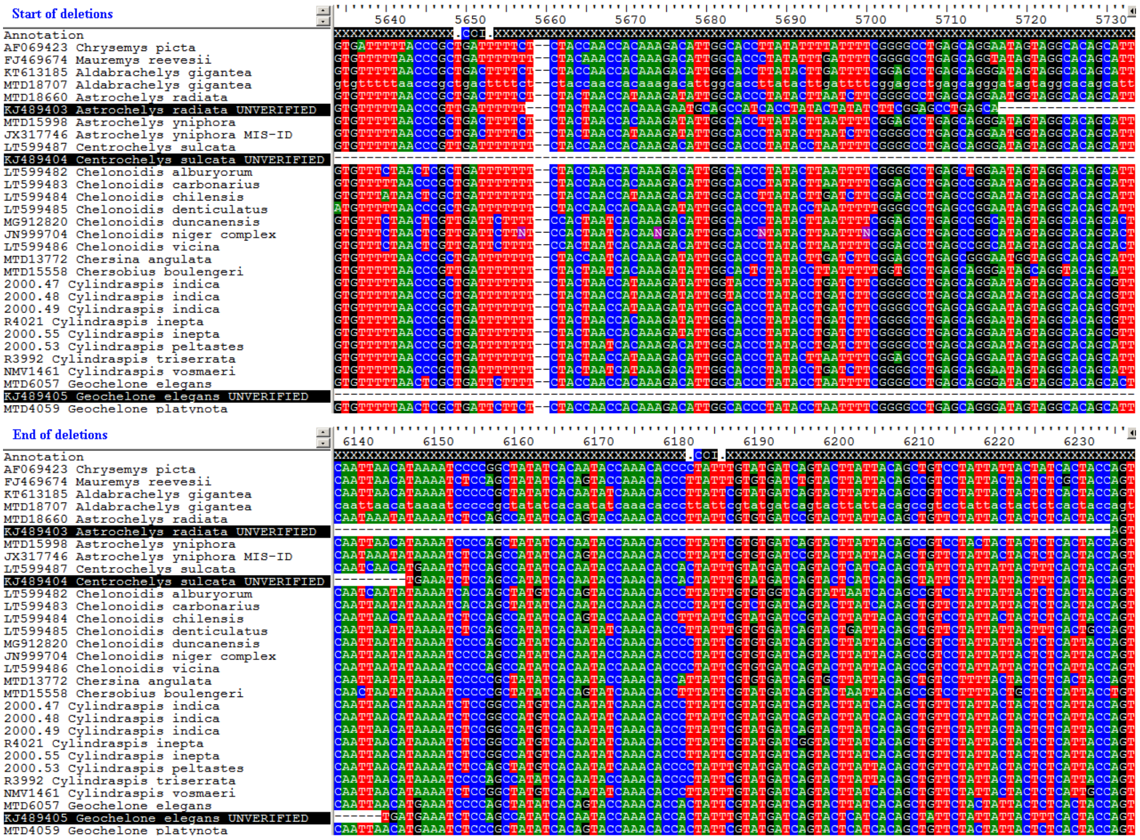


**Figure S2.** Example of assembly artefact in KJ489403–KJ489405. Shown are the start (top) and the end (bottom) of approximately 500-bp-long deletions at the beginning of COI.

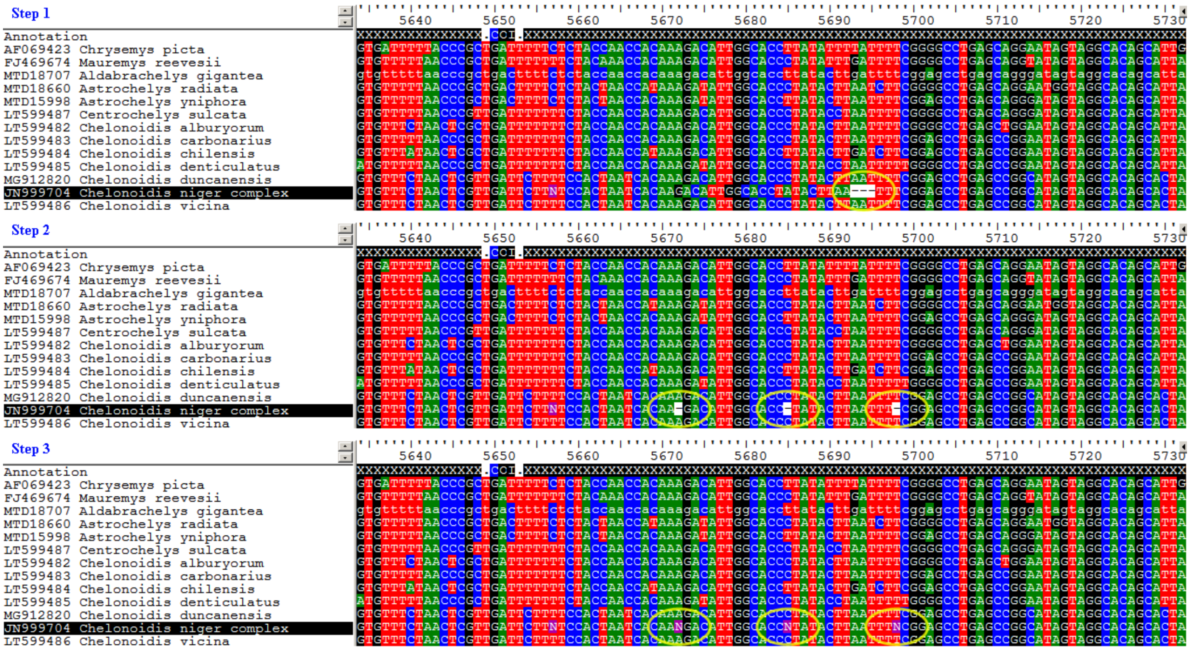

**Figure S3.** Excerpt of the alignment of mitogenomes showing the 5´-end of COI and the manual adjustment made to sequence JN999704 in order to account for a presumable assembly artefact.

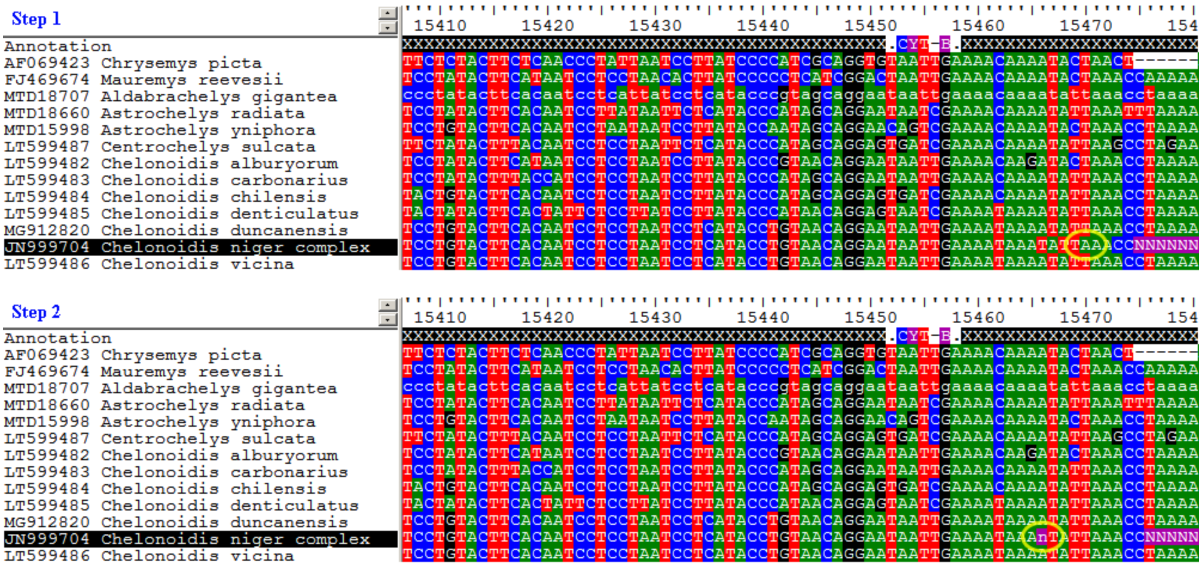

**Figure S4.** Excerpt of the alignment of mitogenomes showing the 3´-end of cyt *b* and the manual adjustment made to sequence JN999704 in order to account for an internal stop codon (TAA, see above) which is a presumable assembly artefact.

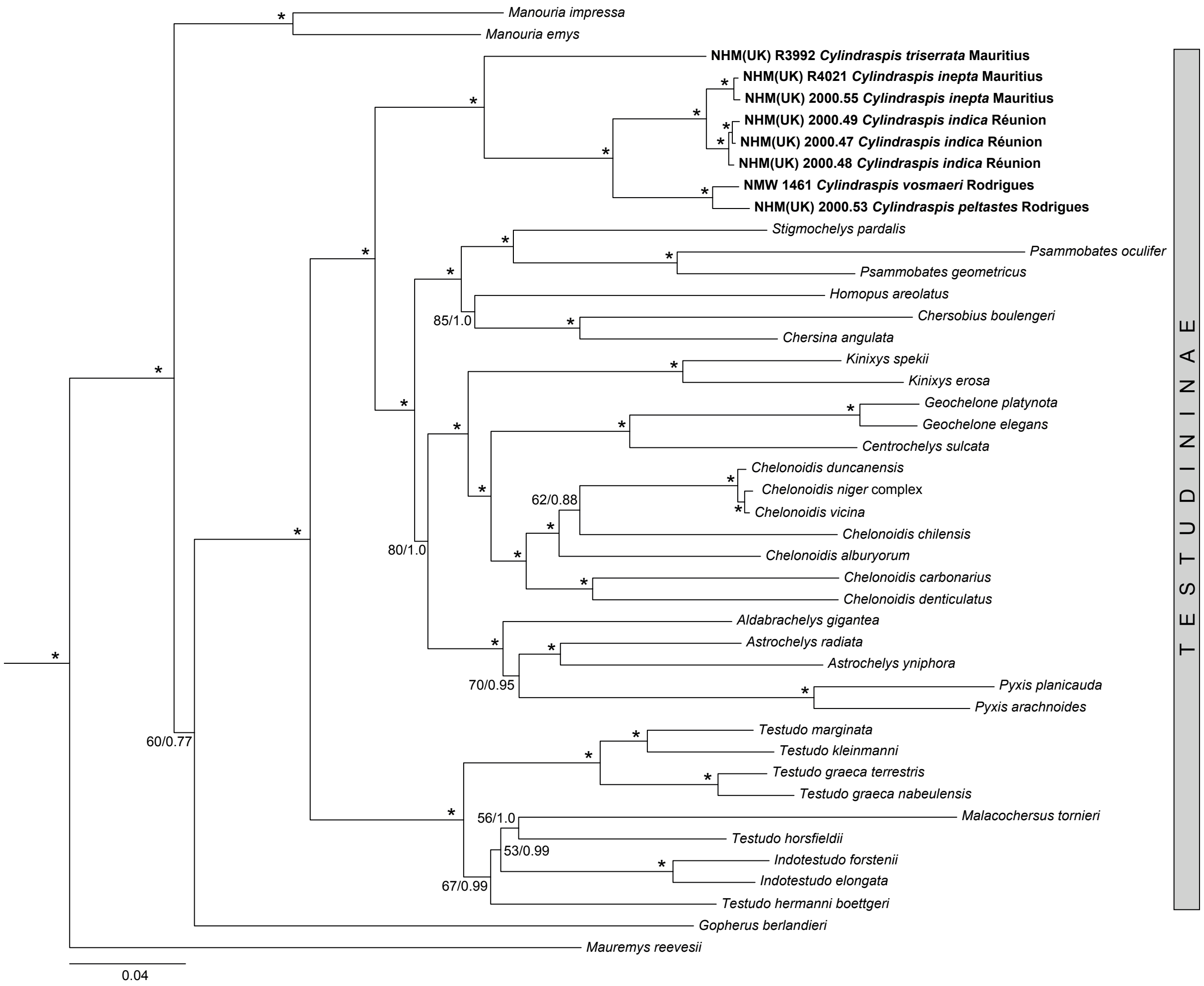


**Figure S5.** Maximum Likelihood tree for *Cylindraspis* and all extant tortoise genera, based on near-complete mitochondrial genomes (up to 15,510 bp). Outgroup used for tree rooting (*Chrysemys picta*) removed for clarity. Numbers at nodes are thorough bootstrap values and posterior probabilities from a Bayesian Inference tree of the same topology. Asterisks indicate maximum support under both approaches.

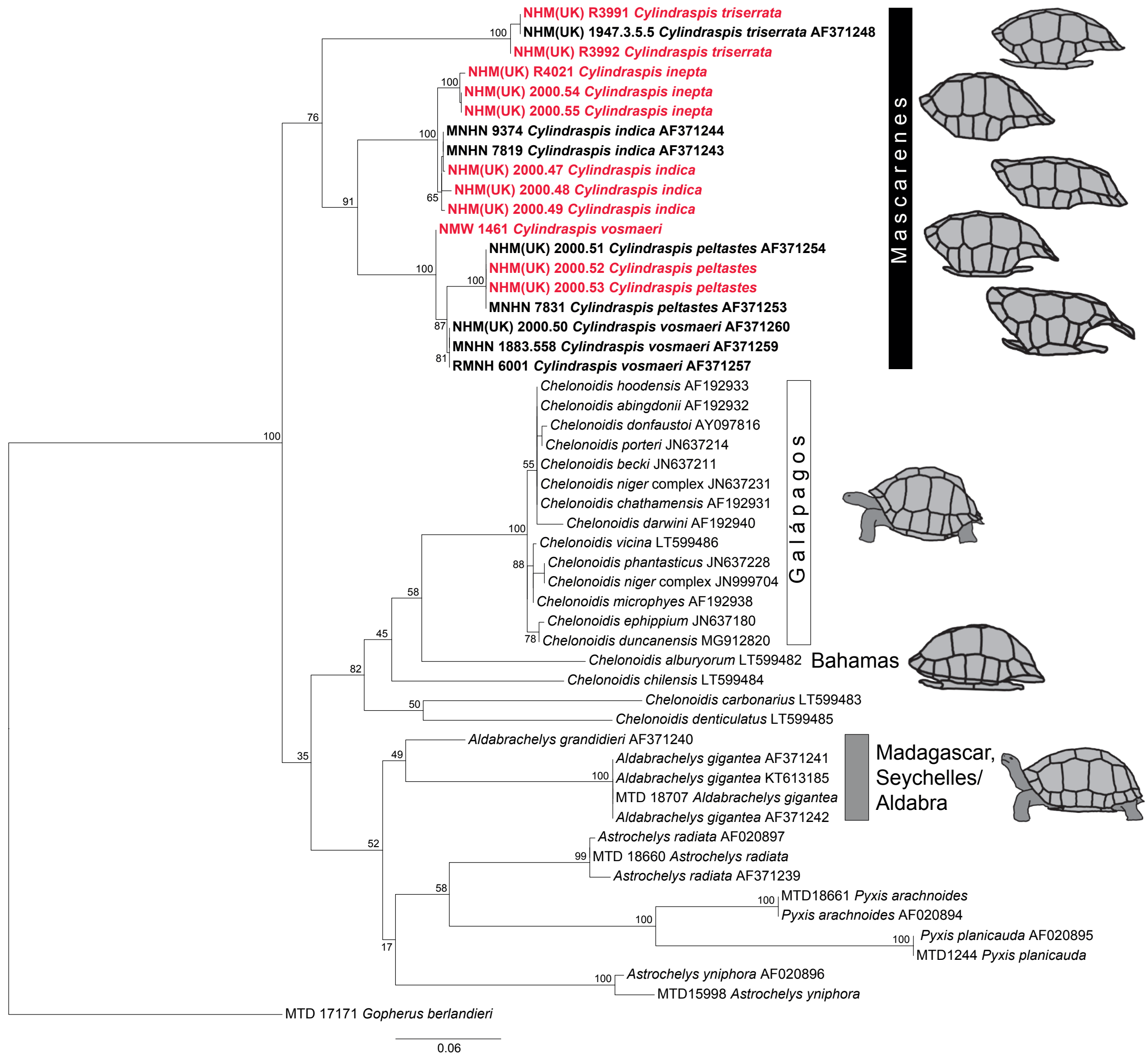


**Figure S6.** Maximum Likelihood tree based on the complete cytochrome *b* gene (up to 1,143 bp) of *Cylindraspis* and selected other testudinine tortoises, rooted with *Gopherus berlandieri*. Included are all genera with giant tortoise species (highlighted by icons) and all taxa from islands in the Western Indian Ocean (Madagascar, Aldabra). Sequences for *Cylindraspis* produced in the present study shown in red; *Cylindraspis* sequences from Austin & Arnold (2001), black. Species names are followed by GenBank/ENA accession numbers; sequences produced for the present study are labelled with MTD numbers. For *Cylindraspis* samples, the collection numbers with acronyms are given; acronyms are: MTD = Museum of Zoology, Senckenberg Dresden; MNHN = Muséum National d'Histoire naturelle, Paris; NHM(UK) = Natural History Museum of the United Kingdom, London; NMW = Natural History Museum, Vienna; RMNH = Naturalis, Leiden. Drawings: Christian Schmidt.

| | | 1 | 2 | 3 | 4 | 5 | 6 | 7 | 8 | 9 | 10 | 11 | 12 | 13 | 14 | 15 | 16 | 17 | 18 | 19 | 20 | 21 | 22 | 23 | 24 |
|---|---|---|---|---|---|---|---|---|---|---|---|---|---|---|---|---|---|---|---|---|---|---|---|---|---|
| 1 | AF069423 Chrysemys picta | — | | | | | | | | | | | | | | | | | | | | | | | |
| 2 | FJ469674 Mauremys reevesii | 0.179 | — | | | | | | | | | | | | | | | | | | | | | | |
| 3 | MTD18707 Aldabrachelys gigantea | 0.171 | 0.146 | — | | | | | | | | | | | | | | | | | | | | | |
| 4 | MTD18660 Astrochelys radiata | 0.175 | 0.149 | 0.088 | — | | | | | | | | | | | | | | | | | | | | |
| 5 | MTD15998 Astrochelys yniphora | 0.177 | 0.152 | 0.095 | 0.088 | — | | | | | | | | | | | | | | | | | | | |
| 6 | LT599487 Centrochelys sulcata | 0.175 | 0.155 | 0.107 | 0.114 | 0.118 | — | | | | | | | | | | | | | | | | | | |
| 7 | LT599482 Chelonoidis alburyorum | 0.173 | 0.150 | 0.099 | 0.102 | 0.107 | 0.100 | — | | | | | | | | | | | | | | | | | |
| 8 | LT599483 Chelonoidis carbonarius | 0.177 | 0.152 | 0.108 | 0.114 | 0.118 | 0.111 | 0.097 | — | | | | | | | | | | | | | | | | |
| 9 | LT599484 Chelonoidis chilensis | 0.177 | 0.152 | 0.106 | 0.109 | 0.116 | 0.108 | 0.092 | 0.104 | — | | | | | | | | | | | | | | | |
| 10 | LT599485 Chelonoidis denticulatus | 0.178 | 0.154 | 0.109 | 0.112 | 0.118 | 0.107 | 0.095 | 0.095 | 0.106 | — | | | | | | | | | | | | | | |
| 11 | MG912820 Chelonoidis duncanensis | 0.171 | 0.147 | 0.097 | 0.102 | 0.106 | 0.098 | 0.076 | 0.095 | 0.085 | 0.094 | — | | | | | | | | | | | | | |
| 12 | JN999704 Chelonoidis niger complex | 0.172 | 0.149 | 0.097 | 0.102 | 0.107 | 0.099 | 0.077 | 0.096 | 0.085 | 0.094 | 0.007 | — | | | | | | | | | | | | |
| 13 | LT599486 Chelonoidis vicina | 0.171 | 0.147 | 0.097 | 0.102 | 0.107 | 0.098 | 0.076 | 0.095 | 0.085 | 0.094 | 0.006 | 0.004 | — | | | | | | | | | | | |
| 14 | MTD13772 Chersina angulata | 0.174 | 0.154 | 0.108 | 0.111 | 0.115 | 0.119 | 0.110 | 0.121 | 0.117 | 0.119 | 0.109 | 0.112 | 0.111 | — | | | | | | | | | | |
| 15 | MTD15558 Chersobius boulengeri | 0.186 | 0.165 | 0.125 | 0.127 | 0.131 | 0.136 | 0.124 | 0.135 | 0.133 | 0.135 | 0.125 | 0.126 | 0.126 | 0.099 | — | | | | | | | | | |
| 16 | NHM(UK) 2000.47 Cylindraspis indica | 0.175 | 0.148 | 0.111 | 0.115 | 0.117 | 0.118 | 0.109 | 0.119 | 0.115 | 0.118 | 0.107 | 0.107 | 0.107 | 0.118 | 0.133 | — | | | | | | | | |
| 17 | NHM(UK) 2000.48 Cylindraspis indica | 0.175 | 0.148 | 0.111 | 0.115 | 0.117 | 0.119 | 0.109 | 0.120 | 0.115 | 0.119 | 0.108 | 0.108 | 0.108 | 0.118 | 0.133 | 0.004 | — | | | | | | | |
| 18 | NHM(UK) 2000.49 Cylindraspis indica | 0.175 | 0.148 | 0.111 | 0.116 | 0.117 | 0.118 | 0.109 | 0.121 | 0.115 | 0.118 | 0.108 | 0.108 | 0.107 | 0.118 | 0.133 | 0.003 | 0.005 | — | | | | | | |
| 19 | NHM(UK) R4021 Cylindraspis inepta | 0.175 | 0.149 | 0.109 | 0.114 | 0.116 | 0.117 | 0.108 | 0.118 | 0.114 | 0.117 | 0.106 | 0.106 | 0.106 | 0.116 | 0.132 | 0.018 | 0.018 | 0.019 | — | | | | | |
| 20 | NHM(UK) 2000.55 Cylindraspis inepta | 0.176 | 0.149 | 0.110 | 0.115 | 0.116 | 0.117 | 0.109 | 0.119 | 0.114 | 0.118 | 0.107 | 0.107 | 0.106 | 0.117 | 0.132 | 0.019 | 0.019 | 0.020 | 0.003 | — | | | | |
| 21 | NHM(UK) 2000.53 Cylindraspis peltastes | 0.175 | 0.146 | 0.111 | 0.114 | 0.119 | 0.118 | 0.113 | 0.119 | 0.120 | 0.118 | 0.111 | 0.110 | 0.110 | 0.119 | 0.133 | 0.062 | 0.062 | 0.062 | 0.061 | 0.062 | — | | | |
| 22 | NHM(UK) R3992 Cylindraspis triserrata | 0.177 | 0.146 | 0.105 | 0.112 | 0.116 | 0.118 | 0.108 | 0.114 | 0.114 | 0.118 | 0.104 | 0.105 | 0.105 | 0.117 | 0.129 | 0.091 | 0.091 | 0.092 | 0.092 | 0.093 | 0.092 | — | | |
| 23 | NMW1461 Cylindraspis vosmaeri | 0.175 | 0.145 | 0.110 | 0.113 | 0.119 | 0.118 | 0.110 | 0.118 | 0.117 | 0.117 | 0.109 | 0.109 | 0.108 | 0.118 | 0.131 | 0.059 | 0.059 | 0.060 | 0.059 | 0.059 | 0.019 | 0.091 | — | |
| 24 | MTD6057 Geochelone elegans | 0.179 | 0.157 | 0.113 | 0.121 | 0.123 | 0.097 | 0.110 | 0.118 | 0.116 | 0.120 | 0.109 | 0.110 | 0.109 | 0.124 | 0.143 | 0.122 | 0.123 | 0.123 | 0.122 | 0.123 | 0.126 | 0.125 | 0.125 | — |
| 25 | MTD4059 Geochelone platynota | 0.182 | 0.157 | 0.116 | 0.122 | 0.125 | 0.096 | 0.109 | 0.119 | 0.117 | 0.120 | 0.109 | 0.110 | 0.109 | 0.125 | 0.142 | 0.124 | 0.125 | 0.125 | 0.124 | 0.125 | 0.127 | 0.128 | 0.126 | 0.032 |
| 26 | MTD17171 Gopherus berlandieri | 0.174 | 0.153 | 0.133 | 0.138 | 0.139 | 0.139 | 0.134 | 0.144 | 0.143 | 0.144 | 0.135 | 0.136 | 0.135 | 0.142 | 0.153 | 0.138 | 0.137 | 0.138 | 0.138 | 0.138 | 0.139 | 0.139 | 0.138 | 0.148 |
| 27 | MTD15479 Homopus areolatus | 0.182 | 0.157 | 0.116 | 0.116 | 0.123 | 0.126 | 0.119 | 0.127 | 0.125 | 0.125 | 0.116 | 0.116 | 0.116 | 0.113 | 0.129 | 0.126 | 0.126 | 0.126 | 0.125 | 0.125 | 0.126 | 0.121 | 0.125 | 0.135 |
| 28 | DQ080043 Indotestudo elongata | 0.177 | 0.152 | 0.121 | 0.125 | 0.128 | 0.126 | 0.119 | 0.127 | 0.127 | 0.128 | 0.118 | 0.119 | 0.119 | 0.127 | 0.140 | 0.125 | 0.125 | 0.125 | 0.124 | 0.125 | 0.127 | 0.123 | 0.125 | 0.137 |
| 29 | DQ080044 Indotestudo forstenii | 0.180 | 0.153 | 0.122 | 0.128 | 0.128 | 0.129 | 0.123 | 0.130 | 0.130 | 0.133 | 0.121 | 0.121 | 0.121 | 0.128 | 0.140 | 0.125 | 0.126 | 0.126 | 0.125 | 0.125 | 0.126 | 0.124 | 0.125 | 0.138 |
| 30 | MTD15816 Kinixys erosa | 0.186 | 0.161 | 0.121 | 0.127 | 0.128 | 0.126 | 0.117 | 0.124 | 0.123 | 0.124 | 0.115 | 0.118 | 0.116 | 0.127 | 0.139 | 0.129 | 0.129 | 0.129 | 0.129 | 0.129 | 0.130 | 0.130 | 0.129 | 0.133 |
| 31 | MTD17037 Kinixys spekii | 0.179 | 0.151 | 0.113 | 0.118 | 0.118 | 0.117 | 0.106 | 0.118 | 0.115 | 0.114 | 0.104 | 0.106 | 0.104 | 0.119 | 0.132 | 0.121 | 0.122 | 0.121 | 0.120 | 0.120 | 0.121 | 0.119 | 0.120 | 0.125 |
| 32 | DQ080042 Malacochersus tornieri | 0.186 | 0.165 | 0.135 | 0.142 | 0.142 | 0.138 | 0.132 | 0.142 | 0.141 | 0.144 | 0.133 | 0.134 | 0.133 | 0.139 | 0.151 | 0.136 | 0.137 | 0.137 | 0.136 | 0.136 | 0.137 | 0.135 | 0.135 | 0.147 |
| 33 | DQ080040 Manouria emys | 0.172 | 0.141 | 0.127 | 0.133 | 0.134 | 0.134 | 0.128 | 0.136 | 0.135 | 0.135 | 0.123 | 0.126 | 0.125 | 0.137 | 0.148 | 0.132 | 0.133 | 0.133 | 0.132 | 0.133 | 0.133 | 0.131 | 0.132 | 0.145 |
| 34 | EF661586 Manouria impressa | 0.174 | 0.143 | 0.131 | 0.134 | 0.133 | 0.135 | 0.132 | 0.137 | 0.139 | 0.138 | 0.130 | 0.131 | 0.131 | 0.138 | 0.149 | 0.134 | 0.135 | 0.135 | 0.134 | 0.134 | 0.135 | 0.133 | 0.134 | 0.145 |
| 35 | MTD13895 Psammobates geometricus | 0.183 | 0.157 | 0.116 | 0.119 | 0.125 | 0.124 | 0.114 | 0.126 | 0.121 | 0.125 | 0.116 | 0.118 | 0.117 | 0.116 | 0.127 | 0.124 | 0.124 | 0.124 | 0.124 | 0.124 | 0.123 | 0.121 | 0.121 | 0.134 |
| 36 | MTD18196 Psammobates oculifer | 0.195 | 0.173 | 0.137 | 0.139 | 0.144 | 0.142 | 0.135 | 0.144 | 0.140 | 0.144 | 0.136 | 0.138 | 0.137 | 0.134 | 0.143 | 0.140 | 0.141 | 0.141 | 0.140 | 0.140 | 0.142 | 0.141 | 0.141 | 0.152 |
| 37 | MTD18661 Pyxis arachnoides | 0.180 | 0.158 | 0.108 | 0.109 | 0.116 | 0.130 | 0.123 | 0.128 | 0.125 | 0.128 | 0.122 | 0.123 | 0.122 | 0.125 | 0.139 | 0.131 | 0.131 | 0.131 | 0.130 | 0.129 | 0.131 | 0.127 | 0.131 | 0.135 |
| 38 | MTD1244 Pyxis planicauda | 0.183 | 0.164 | 0.116 | 0.112 | 0.120 | 0.135 | 0.125 | 0.129 | 0.130 | 0.131 | 0.125 | 0.125 | 0.124 | 0.128 | 0.138 | 0.137 | 0.138 | 0.138 | 0.137 | 0.136 | 0.134 | 0.131 | 0.135 | 0.143 |
| 39 | MTD16076 Stigmochelys pardalis | 0.175 | 0.150 | 0.107 | 0.109 | 0.114 | 0.114 | 0.104 | 0.116 | 0.113 | 0.117 | 0.108 | 0.108 | 0.108 | 0.106 | 0.123 | 0.114 | 0.114 | 0.115 | 0.114 | 0.114 | 0.115 | 0.113 | 0.114 | 0.118 |
| 40 | DQ080049 Testudo graeca nabeulensis | 0.179 | 0.155 | 0.129 | 0.131 | 0.134 | 0.135 | 0.129 | 0.134 | 0.134 | 0.135 | 0.126 | 0.126 | 0.126 | 0.133 | 0.146 | 0.130 | 0.130 | 0.130 | 0.129 | 0.130 | 0.132 | 0.126 | 0.131 | 0.139 |
| 41 | DQ080050 Testudo graeca terrestris | 0.176 | 0.155 | 0.125 | 0.128 | 0.129 | 0.132 | 0.124 | 0.130 | 0.132 | 0.132 | 0.123 | 0.123 | 0.123 | 0.128 | 0.142 | 0.125 | 0.125 | 0.126 | 0.124 | 0.125 | 0.126 | 0.122 | 0.125 | 0.136 |
| 42 | DQ080046 Testudo hermanni boettgeri | 0.180 | 0.152 | 0.122 | 0.125 | 0.128 | 0.130 | 0.121 | 0.128 | 0.129 | 0.129 | 0.117 | 0.119 | 0.118 | 0.126 | 0.139 | 0.126 | 0.126 | 0.126 | 0.126 | 0.127 | 0.127 | 0.125 | 0.126 | 0.136 |
| 43 | DQ080045 Testudo horsfieldii | 0.177 | 0.155 | 0.122 | 0.127 | 0.129 | 0.130 | 0.123 | 0.127 | 0.132 | 0.130 | 0.118 | 0.120 | 0.120 | 0.129 | 0.140 | 0.125 | 0.126 | 0.126 | 0.126 | 0.126 | 0.126 | 0.123 | 0.125 | 0.136 |
| 44 | DQ080048 Testudo kleinmanni | 0.176 | 0.155 | 0.126 | 0.128 | 0.133 | 0.132 | 0.126 | 0.131 | 0.130 | 0.132 | 0.124 | 0.124 | 0.124 | 0.130 | 0.143 | 0.127 | 0.127 | 0.127 | 0.126 | 0.127 | 0.126 | 0.126 | 0.126 | 0.138 |
| 45 | DQ080047 Testudo marginata | 0.175 | 0.154 | 0.123 | 0.128 | 0.131 | 0.130 | 0.123 | 0.133 | 0.130 | 0.133 | 0.122 | 0.122 | 0.123 | 0.127 | 0.143 | 0.125 | 0.125 | 0.125 | 0.123 | 0.124 | 0.126 | 0.124 | 0.124 | 0.138 |

**Figure S7.** Uncorrected *p* distances of near-complete mitogenomes, based on 15,510 aligned sites (mitogenomes).

| | | 25 | 26 | 27 | 28 | 29 | 30 | 31 | 32 | 33 | 34 | 35 | 36 | 37 | 38 | 39 | 40 | 41 | 42 | 43 | 44 | 45 |
|---|---|---|---|---|---|---|---|---|---|---|---|---|---|---|---|---|---|---|---|---|---|---|
| 1 | AF069423 Chrysemys picta | | | | | | | | | | | | | | | | | | | | | |
| 2 | FJ469674 Mauremys reevesii | | | | | | | | | | | | | | | | | | | | | |
| 3 | MTD18707 Aldabrachelys gigantea | | | | | | | | | | | | | | | | | | | | | |
| 4 | MTD18660 Astrochelys radiata | | | | | | | | | | | | | | | | | | | | | |
| 5 | MTD15998 Astrochelys yniphora | | | | | | | | | | | | | | | | | | | | | |
| 6 | LT599487 Centrochelys sulcata | | | | | | | | | | | | | | | | | | | | | |
| 7 | LT599482 Chelonoidis alburyorum | | | | | | | | | | | | | | | | | | | | | |
| 8 | LT599483 Chelonoidis carbonarius | | | | | | | | | | | | | | | | | | | | | |
| 9 | LT599484 Chelonoidis chilensis | | | | | | | | | | | | | | | | | | | | | |
| 10 | LT599485 Chelonoidis denticulatus | | | | | | | | | | | | | | | | | | | | | |
| 11 | MG912820 Chelonoidis duncanensis | | | | | | | | | | | | | | | | | | | | | |
| 12 | JN999704 Chelonoidis niger complex | | | | | | | | | | | | | | | | | | | | | |
| 13 | LT599486 Chelonoidis vicina | | | | | | | | | | | | | | | | | | | | | |
| 14 | MTD13772 Chersina angulata | | | | | | | | | | | | | | | | | | | | | |
| 15 | MTD15558 Chersobius boulengeri | | | | | | | | | | | | | | | | | | | | | |
| 16 | NHM(UK) 2000.47 Cylindraspis indica | | | | | | | | | | | | | | | | | | | | | |
| 17 | NHM(UK) 2000.48 Cylindraspis indica | | | | | | | | | | | | | | | | | | | | | |
| 18 | NHM(UK) 2000.49 Cylindraspis indica | | | | | | | | | | | | | | | | | | | | | |
| 19 | NHM(UK) R4021 Cylindraspis inepta | | | | | | | | | | | | | | | | | | | | | |
| 20 | NHM(UK) 2000.55 Cylindraspis inepta | | | | | | | | | | | | | | | | | | | | | |
| 21 | NHM(UK) 2000.53 Cylindraspis peltastes | | | | | | | | | | | | | | | | | | | | | |
| 22 | NHM(UK) R3992 Cylindraspis triserrata | | | | | | | | | | | | | | | | | | | | | |
| 23 | NMW1461 Cylindraspis vosmaeri | | | | | | | | | | | | | | | | | | | | | |
| 24 | MTD6057 Geochelone elegans | | | | | | | | | | | | | | | | | | | | | |
| 25 | MTD4059 Geochelone platynota | — | | | | | | | | | | | | | | | | | | | | |
| 26 | MTD17171 Gopherus berlandieri | 0.147 | — | | | | | | | | | | | | | | | | | | | |
| 27 | MTD15479 Homopus areolatus | 0.135 | 0.145 | — | | | | | | | | | | | | | | | | | | |
| 28 | DQ080043 Indotestudo elongata | 0.137 | 0.141 | 0.134 | — | | | | | | | | | | | | | | | | | |
| 29 | DQ080044 Indotestudo forstenii | 0.138 | 0.142 | 0.136 | 0.045 | — | | | | | | | | | | | | | | | | |
| 30 | MTD15816 Kinixys erosa | 0.134 | 0.152 | 0.133 | 0.139 | 0.141 | — | | | | | | | | | | | | | | | |
| 31 | MTD17037 Kinixys spekii | 0.125 | 0.142 | 0.124 | 0.128 | 0.132 | 0.080 | — | | | | | | | | | | | | | | |
| 32 | DQ080042 Malacochersus tornieri | 0.147 | 0.153 | 0.144 | 0.113 | 0.114 | 0.152 | 0.142 | — | | | | | | | | | | | | | |
| 33 | DQ080040 Manouria emys | 0.144 | 0.129 | 0.141 | 0.134 | 0.138 | 0.145 | 0.137 | 0.147 | — | | | | | | | | | | | | |
| 34 | EF661586 Manouria impressa | 0.145 | 0.132 | 0.143 | 0.132 | 0.135 | 0.149 | 0.138 | 0.145 | 0.087 | — | | | | | | | | | | | |
| 35 | MTD13895 Psammobates geometricus | 0.134 | 0.146 | 0.120 | 0.134 | 0.134 | 0.134 | 0.126 | 0.145 | 0.138 | 0.142 | — | | | | | | | | | | |
| 36 | MTD18196 Psammobates oculifer | 0.152 | 0.160 | 0.137 | 0.149 | 0.152 | 0.149 | 0.143 | 0.159 | 0.157 | 0.160 | 0.102 | — | | | | | | | | | |
| 37 | MTD18661 Pyxis arachnoides | 0.137 | 0.149 | 0.133 | 0.137 | 0.139 | 0.135 | 0.129 | 0.147 | 0.145 | 0.147 | 0.133 | 0.149 | — | | | | | | | | |
| 38 | MTD1244 Pyxis planicauda | 0.142 | 0.151 | 0.136 | 0.142 | 0.143 | 0.139 | 0.131 | 0.152 | 0.149 | 0.150 | 0.137 | 0.153 | 0.071 | — | | | | | | | |
| 39 | MTD16076 Stigmochelys pardalis | 0.121 | 0.141 | 0.114 | 0.127 | 0.129 | 0.124 | 0.118 | 0.140 | 0.133 | 0.135 | 0.105 | 0.127 | 0.126 | 0.130 | — | | | | | | |
| 40 | DQ080049 Testudo graeca nabeulensis | 0.142 | 0.147 | 0.138 | 0.107 | 0.110 | 0.145 | 0.135 | 0.125 | 0.140 | 0.141 | 0.141 | 0.155 | 0.143 | 0.145 | 0.133 | — | | | | | |
| 41 | DQ080050 Testudo graeca terrestris | 0.138 | 0.144 | 0.136 | 0.102 | 0.106 | 0.141 | 0.130 | 0.121 | 0.137 | 0.138 | 0.137 | 0.150 | 0.139 | 0.142 | 0.128 | 0.035 | — | | | | |
| 42 | DQ080046 Testudo hermanni boettgeri | 0.138 | 0.141 | 0.132 | 0.094 | 0.096 | 0.138 | 0.130 | 0.113 | 0.133 | 0.133 | 0.134 | 0.148 | 0.138 | 0.142 | 0.125 | 0.101 | 0.099 | — | | | |
| 43 | DQ080045 Testudo horsfieldii | 0.138 | 0.142 | 0.136 | 0.094 | 0.095 | 0.140 | 0.132 | 0.114 | 0.134 | 0.135 | 0.134 | 0.148 | 0.140 | 0.144 | 0.127 | 0.106 | 0.102 | 0.089 | — | | |
| 44 | DQ080048 Testudo kleinmanni | 0.138 | 0.147 | 0.140 | 0.105 | 0.108 | 0.141 | 0.133 | 0.122 | 0.135 | 0.138 | 0.137 | 0.152 | 0.140 | 0.142 | 0.128 | 0.080 | 0.074 | 0.102 | 0.103 | — | |
| 45 | DQ080047 Testudo marginata | 0.138 | 0.145 | 0.136 | 0.100 | 0.102 | 0.139 | 0.132 | 0.119 | 0.132 | 0.137 | 0.136 | 0.149 | 0.139 | 0.144 | 0.129 | 0.075 | 0.070 | 0.100 | 0.100 | 0.058 | — |

**Figure S7.** *Continued.*

| | | 1 | 2 | 3 | 4 | 5 | 6 | 7 | 8 | 9 | 10 | 11 | 12 | 13 | 14 | 15 | 16 | 17 | 18 | 19 | 20 | 21 | 22 | 23 | 24 | 25 | 26 | 27 | 28 | 29 |
|---|---|---|---|---|---|---|---|---|---|---|---|---|---|---|---|---|---|---|---|---|---|---|---|---|---|---|---|---|---|---|
| 1 | MTD17171 Gopherus berlandieri | — | | | | | | | | | | | | | | | | | | | | | | | | | | | | |
| 2 | MTD18707 Aldabrachelys gigantea | 0.127 | — | | | | | | | | | | | | | | | | | | | | | | | | | | | |
| 3 | AF371241 Aldabrachelys gigantea | 0.111 | 0.000 | — | | | | | | | | | | | | | | | | | | | | | | | | | | |
| 4 | AF371242 Aldabrachelys gigantea | 0.123 | 0.000 | 0.000 | — | | | | | | | | | | | | | | | | | | | | | | | | | |
| 5 | KT613185 Aldabrachelys gigantea | 0.127 | 0.000 | 0.000 | 0.000 | — | | | | | | | | | | | | | | | | | | | | | | | | |
| 6 | AF371240 Aldabrachelys grandidieri | 0.106 | 0.062 | 0.062 | 0.062 | 0.062 | — | | | | | | | | | | | | | | | | | | | | | | | |
| 7 | MTD18660 Astrochelys radiata | 0.150 | 0.105 | 0.081 | 0.105 | 0.105 | 0.057 | — | | | | | | | | | | | | | | | | | | | | | | |
| 8 | AF020897 Astrochelys radiata | 0.130 | 0.080 | 0.080 | 0.080 | 0.080 | 0.055 | 0.003 | — | | | | | | | | | | | | | | | | | | | | | |
| 9 | AF371239 Astrochelys radiata | 0.133 | 0.086 | 0.086 | 0.086 | 0.086 | 0.062 | 0.010 | 0.010 | — | | | | | | | | | | | | | | | | | | | | |
| 10 | MTD15998 Astrochelys yniphora | 0.126 | 0.101 | 0.094 | 0.102 | 0.101 | 0.079 | 0.110 | 0.096 | 0.096 | — | | | | | | | | | | | | | | | | | | | |
| 11 | AF020896 Astrochelys yniphora | 0.120 | 0.094 | 0.094 | 0.094 | 0.094 | 0.073 | 0.081 | 0.083 | 0.086 | 0.021 | — | | | | | | | | | | | | | | | | | | |
| 12 | LT599482 Chelonoidis alburyorum | 0.131 | 0.115 | 0.099 | 0.115 | 0.115 | 0.074 | 0.107 | 0.098 | 0.101 | 0.117 | 0.094 | — | | | | | | | | | | | | | | | | | |
| 13 | LT599483 Chelonoidis carbonarius | 0.142 | 0.109 | 0.109 | 0.116 | 0.109 | 0.097 | 0.122 | 0.104 | 0.106 | 0.126 | 0.128 | 0.113 | — | | | | | | | | | | | | | | | | |
| 14 | LT599484 Chelonoidis chilensis | 0.136 | 0.110 | 0.109 | 0.116 | 0.110 | 0.092 | 0.108 | 0.098 | 0.104 | 0.111 | 0.115 | 0.100 | 0.112 | — | | | | | | | | | | | | | | | |
| 15 | LT599485 Chelonoidis denticulatus | 0.144 | 0.121 | 0.091 | 0.109 | 0.121 | 0.069 | 0.125 | 0.085 | 0.091 | 0.129 | 0.104 | 0.100 | 0.107 | 0.116 | — | | | | | | | | | | | | | | |
| 16 | AF192932 Chelonoidis abingdonii | 0.108 | 0.088 | 0.086 | 0.088 | 0.088 | 0.079 | 0.086 | 0.080 | 0.086 | 0.086 | 0.086 | 0.074 | 0.103 | 0.076 | 0.076 | — | | | | | | | | | | | | | |
| 17 | JN637211 Chelonoidis becki | 0.108 | 0.089 | 0.086 | 0.089 | 0.089 | 0.079 | 0.087 | 0.080 | 0.086 | 0.087 | 0.086 | 0.075 | 0.103 | 0.077 | 0.077 | 0.000 | — | | | | | | | | | | | | |
| 18 | AF192931 Chelonoidis chathamensis | 0.108 | 0.088 | 0.086 | 0.088 | 0.088 | 0.079 | 0.086 | 0.080 | 0.086 | 0.086 | 0.086 | 0.074 | 0.103 | 0.076 | 0.076 | 0.000 | 0.000 | — | | | | | | | | | | | |
| 19 | AF192940 Chelonoidis darwini | 0.110 | 0.086 | 0.084 | 0.086 | 0.086 | 0.072 | 0.083 | 0.080 | 0.089 | 0.078 | 0.076 | 0.071 | 0.100 | 0.083 | 0.076 | 0.012 | 0.012 | 0.012 | — | | | | | | | | | | |
| 20 | AY097816 Chelonoidis donfaustoi | 0.111 | 0.089 | 0.084 | 0.089 | 0.089 | 0.077 | 0.087 | 0.080 | 0.084 | 0.087 | 0.086 | 0.075 | 0.103 | 0.077 | 0.077 | 0.002 | 0.005 | 0.002 | 0.015 | — | | | | | | | | | |
| 21 | MG912820 Chelonoidis duncanensis | 0.134 | 0.106 | 0.094 | 0.105 | 0.106 | 0.084 | 0.122 | 0.091 | 0.094 | 0.107 | 0.091 | 0.090 | 0.107 | 0.089 | 0.102 | 0.012 | 0.014 | 0.012 | 0.025 | 0.014 | — | | | | | | | | |
| 22 | JN637180 Chelonoidis ephippium | 0.115 | 0.096 | 0.094 | 0.096 | 0.096 | 0.084 | 0.094 | 0.091 | 0.094 | 0.089 | 0.091 | 0.082 | 0.101 | 0.084 | 0.079 | 0.012 | 0.012 | 0.012 | 0.025 | 0.012 | 0.002 | — | | | | | | | |
| 23 | AF192933 Chelonoidis hoodensis | 0.108 | 0.088 | 0.086 | 0.088 | 0.088 | 0.079 | 0.086 | 0.080 | 0.086 | 0.086 | 0.086 | 0.074 | 0.103 | 0.076 | 0.076 | 0.000 | 0.000 | 0.000 | 0.012 | 0.002 | 0.012 | 0.012 | — | | | | | | |
| 24 | AF192938 Chelonoidis microphyes | 0.110 | 0.091 | 0.089 | 0.091 | 0.091 | 0.084 | 0.088 | 0.083 | 0.089 | 0.093 | 0.094 | 0.081 | 0.100 | 0.083 | 0.078 | 0.007 | 0.007 | 0.007 | 0.020 | 0.010 | 0.015 | 0.015 | 0.007 | — | | | | | |
| 25 | JN637231 Chelonoidis niger complex | 0.107 | 0.088 | 0.086 | 0.088 | 0.088 | 0.079 | 0.085 | 0.080 | 0.086 | 0.085 | 0.086 | 0.073 | 0.102 | 0.076 | 0.076 | 0.000 | 0.000 | 0.000 | 0.012 | 0.002 | 0.012 | 0.012 | 0.000 | 0.007 | — | | | | |
| 26 | JN999704 Chelonoidis niger complex | 0.134 | 0.110 | 0.086 | 0.104 | 0.110 | 0.082 | 0.124 | 0.080 | 0.086 | 0.113 | 0.096 | 0.096 | 0.112 | 0.095 | 0.108 | 0.010 | 0.012 | 0.010 | 0.022 | 0.017 | 0.016 | 0.019 | 0.010 | 0.002 | 0.010 | — | | | |
| 27 | JN637228 Chelonoidis phantasticus | 0.107 | 0.088 | 0.086 | 0.088 | 0.088 | 0.082 | 0.085 | 0.080 | 0.086 | 0.095 | 0.096 | 0.083 | 0.098 | 0.085 | 0.076 | 0.010 | 0.010 | 0.010 | 0.022 | 0.012 | 0.017 | 0.017 | 0.010 | 0.002 | 0.010 | 0.000 | — | | |
| 28 | JN637214 Chelonoidis porteri | 0.108 | 0.087 | 0.084 | 0.087 | 0.087 | 0.077 | 0.084 | 0.080 | 0.084 | 0.084 | 0.086 | 0.072 | 0.101 | 0.075 | 0.075 | 0.002 | 0.002 | 0.002 | 0.015 | 0.002 | 0.012 | 0.010 | 0.002 | 0.010 | 0.002 | 0.014 | 0.012 | — | |
| 29 | LT599486 Chelonoidis vicina | 0.130 | 0.107 | 0.086 | 0.104 | 0.107 | 0.082 | 0.121 | 0.083 | 0.086 | 0.108 | 0.094 | 0.091 | 0.108 | 0.092 | 0.107 | 0.010 | 0.012 | 0.010 | 0.022 | 0.017 | 0.011 | 0.019 | 0.010 | 0.002 | 0.010 | 0.008 | 0.005 | 0.014 | — |
| 30 | MTD18661 Pyxis arachnoides | 0.155 | 0.107 | 0.109 | 0.109 | 0.107 | 0.099 | 0.109 | 0.096 | 0.109 | 0.122 | 0.117 | 0.130 | 0.139 | 0.117 | 0.134 | 0.123 | 0.123 | 0.123 | 0.110 | 0.123 | 0.135 | 0.130 | 0.123 | 0.120 | 0.122 | 0.137 | 0.122 | 0.120 | 0.134 |
| 31 | AF020894 Pyxis arachnoides | 0.161 | 0.106 | 0.106 | 0.106 | 0.106 | 0.096 | 0.098 | 0.096 | 0.106 | 0.122 | 0.117 | 0.124 | 0.145 | 0.124 | 0.135 | 0.122 | 0.122 | 0.122 | 0.111 | 0.122 | 0.132 | 0.132 | 0.122 | 0.119 | 0.122 | 0.122 | 0.122 | 0.122 | 0.119 |
| 32 | MTD1244 Pyxis planicauda | 0.154 | 0.121 | 0.114 | 0.111 | 0.121 | 0.111 | 0.122 | 0.104 | 0.111 | 0.136 | 0.130 | 0.146 | 0.138 | 0.136 | 0.148 | 0.123 | 0.123 | 0.123 | 0.118 | 0.127 | 0.148 | 0.130 | 0.123 | 0.125 | 0.122 | 0.149 | 0.122 | 0.125 | 0.145 |
| 33 | AF020895 Pyxis planicauda | 0.130 | 0.109 | 0.109 | 0.109 | 0.109 | 0.104 | 0.106 | 0.104 | 0.106 | 0.132 | 0.130 | 0.119 | 0.132 | 0.127 | 0.124 | 0.111 | 0.111 | 0.111 | 0.109 | 0.111 | 0.117 | 0.117 | 0.111 | 0.114 | 0.111 | 0.111 | 0.111 | 0.111 | 0.114 |
| 34 | NHM(UK) 2000.47 Cylindraspis indica | 0.126 | 0.101 | 0.089 | 0.095 | 0.101 | 0.072 | 0.110 | 0.093 | 0.094 | 0.100 | 0.096 | 0.103 | 0.112 | 0.101 | 0.110 | 0.083 | 0.084 | 0.083 | 0.086 | 0.084 | 0.088 | 0.087 | 0.083 | 0.081 | 0.083 | 0.092 | 0.078 | 0.082 | 0.089 |
| 35 | NHM(UK) 2000.48 Cylindraspis indica | 0.125 | 0.102 | 0.091 | 0.098 | 0.102 | 0.077 | 0.114 | 0.096 | 0.096 | 0.103 | 0.099 | 0.107 | 0.115 | 0.099 | 0.117 | 0.076 | 0.077 | 0.076 | 0.078 | 0.077 | 0.090 | 0.084 | 0.076 | 0.078 | 0.076 | 0.096 | 0.080 | 0.075 | 0.091 |
| 36 | NHM(UK) 2000.49 Cylindraspis indica | 0.128 | 0.102 | 0.094 | 0.098 | 0.102 | 0.077 | 0.112 | 0.098 | 0.099 | 0.100 | 0.096 | 0.102 | 0.115 | 0.098 | 0.112 | 0.078 | 0.079 | 0.078 | 0.081 | 0.079 | 0.086 | 0.082 | 0.078 | 0.076 | 0.078 | 0.091 | 0.078 | 0.077 | 0.087 |
| 37 | AF371243 Cylindraspis indica | 0.114 | 0.091 | 0.091 | 0.091 | 0.091 | 0.074 | 0.096 | 0.096 | 0.096 | 0.084 | 0.094 | 0.094 | 0.111 | 0.099 | 0.091 | 0.081 | 0.081 | 0.081 | 0.084 | 0.079 | 0.084 | 0.084 | 0.081 | 0.079 | 0.081 | 0.081 | 0.081 | 0.079 | 0.077 |
| 38 | AF371244 Cylindraspis indica | 0.115 | 0.092 | 0.092 | 0.092 | 0.092 | 0.075 | 0.097 | 0.096 | 0.097 | 0.085 | 0.094 | 0.095 | 0.112 | 0.100 | 0.092 | 0.082 | 0.082 | 0.082 | 0.085 | 0.080 | 0.085 | 0.085 | 0.082 | 0.080 | 0.082 | 0.082 | 0.082 | 0.080 | 0.077 |
| 39 | NHM(UK) R4021 Cylindraspis inepta | 0.124 | 0.101 | 0.094 | 0.095 | 0.101 | 0.077 | 0.115 | 0.098 | 0.099 | 0.101 | 0.096 | 0.102 | 0.111 | 0.100 | 0.113 | 0.078 | 0.079 | 0.078 | 0.081 | 0.079 | 0.086 | 0.082 | 0.078 | 0.071 | 0.078 | 0.090 | 0.073 | 0.077 | 0.085 |
| 40 | NHM(UK) 2000.54 Cylindraspis inepta | 0.122 | 0.100 | 0.089 | 0.094 | 0.100 | 0.072 | 0.111 | 0.093 | 0.094 | 0.097 | 0.091 | 0.101 | 0.111 | 0.098 | 0.111 | 0.074 | 0.075 | 0.074 | 0.076 | 0.075 | 0.082 | 0.077 | 0.074 | 0.066 | 0.073 | 0.086 | 0.068 | 0.072 | 0.081 |
| 41 | NHM(UK) 2000.55 Cylindraspis inepta | 0.122 | 0.100 | 0.089 | 0.094 | 0.100 | 0.072 | 0.111 | 0.093 | 0.094 | 0.097 | 0.091 | 0.101 | 0.111 | 0.098 | 0.111 | 0.074 | 0.075 | 0.074 | 0.076 | 0.075 | 0.082 | 0.077 | 0.074 | 0.066 | 0.073 | 0.086 | 0.068 | 0.072 | 0.081 |
| 42 | NHM(UK) R3991 Cylindraspis triserrata | 0.124 | 0.109 | 0.089 | 0.098 | 0.109 | 0.092 | 0.114 | 0.093 | 0.099 | 0.109 | 0.096 | 0.113 | 0.108 | 0.108 | 0.122 | 0.066 | 0.067 | 0.066 | 0.069 | 0.072 | 0.097 | 0.079 | 0.066 | 0.069 | 0.066 | 0.101 | 0.071 | 0.070 | 0.096 |
| 43 | NHM(UK) R3992 Cylindraspis triserrata | 0.122 | 0.106 | 0.089 | 0.097 | 0.106 | 0.092 | 0.113 | 0.093 | 0.099 | 0.111 | 0.096 | 0.113 | 0.108 | 0.108 | 0.122 | 0.066 | 0.067 | 0.066 | 0.069 | 0.072 | 0.097 | 0.079 | 0.066 | 0.069 | 0.066 | 0.101 | 0.071 | 0.070 | 0.096 |
| 44 | AF371248 Cylindraspis triserrata | 0.121 | 0.089 | 0.089 | 0.089 | 0.089 | 0.092 | 0.099 | 0.093 | 0.099 | 0.096 | 0.096 | 0.106 | 0.106 | 0.091 | 0.109 | 0.067 | 0.067 | 0.067 | 0.069 | 0.069 | 0.079 | 0.079 | 0.067 | 0.069 | 0.067 | 0.072 | 0.072 | 0.069 | 0.067 |
| 45 | NHM(UK) 2000.52 Cylindraspis peltastes | 0.122 | 0.103 | 0.077 | 0.097 | 0.103 | 0.069 | 0.112 | 0.087 | 0.091 | 0.109 | 0.093 | 0.111 | 0.124 | 0.103 | 0.120 | 0.084 | 0.088 | 0.084 | 0.082 | 0.083 | 0.104 | 0.096 | 0.084 | 0.087 | 0.084 | 0.105 | 0.084 | 0.085 | 0.101 |
| 46 | NHM(UK) 2000.53 Cylindraspis peltastes | 0.128 | 0.105 | 0.086 | 0.100 | 0.105 | 0.069 | 0.115 | 0.091 | 0.094 | 0.109 | 0.091 | 0.112 | 0.126 | 0.107 | 0.118 | 0.083 | 0.087 | 0.083 | 0.081 | 0.082 | 0.106 | 0.094 | 0.083 | 0.086 | 0.083 | 0.106 | 0.083 | 0.084 | 0.103 |
| 47 | AF371253 Cylindraspis peltastes | 0.111 | 0.086 | 0.086 | 0.086 | 0.086 | 0.069 | 0.089 | 0.091 | 0.094 | 0.089 | 0.091 | 0.094 | 0.123 | 0.094 | 0.104 | 0.084 | 0.084 | 0.084 | 0.081 | 0.081 | 0.091 | 0.091 | 0.084 | 0.086 | 0.084 | 0.084 | 0.084 | 0.081 | 0.084 |
| 48 | AF371254 Cylindraspis peltastes | 0.111 | 0.086 | 0.086 | 0.086 | 0.086 | 0.069 | 0.089 | 0.091 | 0.094 | 0.089 | 0.091 | 0.094 | 0.123 | 0.094 | 0.104 | 0.084 | 0.084 | 0.084 | 0.081 | 0.081 | 0.091 | 0.091 | 0.084 | 0.086 | 0.084 | 0.084 | 0.084 | 0.081 | 0.084 |
| 49 | NMW1461 Cylindraspis vosmaeri | 0.127 | 0.098 | 0.084 | 0.094 | 0.098 | 0.077 | 0.108 | 0.088 | 0.091 | 0.108 | 0.104 | 0.104 | 0.117 | 0.096 | 0.110 | 0.081 | 0.084 | 0.081 | 0.083 | 0.079 | 0.095 | 0.091 | 0.081 | 0.083 | 0.080 | 0.096 | 0.080 | 0.082 | 0.093 |
| 50 | AF371257 Cylindraspis vosmaeri | 0.127 | 0.087 | 0.087 | 0.087 | 0.087 | 0.080 | 0.090 | 0.091 | 0.095 | 0.100 | 0.102 | 0.102 | 0.130 | 0.090 | 0.105 | 0.090 | 0.090 | 0.090 | 0.087 | 0.087 | 0.097 | 0.097 | 0.090 | 0.092 | 0.090 | 0.090 | 0.090 | 0.087 | 0.090 |
| 51 | AF371259 Cylindraspis vosmaeri | 0.126 | 0.086 | 0.086 | 0.086 | 0.086 | 0.079 | 0.089 | 0.091 | 0.094 | 0.099 | 0.102 | 0.101 | 0.128 | 0.089 | 0.104 | 0.089 | 0.089 | 0.089 | 0.086 | 0.086 | 0.096 | 0.096 | 0.089 | 0.091 | 0.089 | 0.089 | 0.089 | 0.086 | 0.089 |
| 52 | AF371260 Cylindraspis vosmaeri | 0.126 | 0.086 | 0.086 | 0.086 | 0.086 | 0.079 | 0.089 | 0.091 | 0.094 | 0.099 | 0.102 | 0.101 | 0.128 | 0.089 | 0.104 | 0.089 | 0.089 | 0.089 | 0.086 | 0.086 | 0.096 | 0.096 | 0.089 | 0.091 | 0.089 | 0.089 | 0.089 | 0.086 | 0.089 |

**Figure S8.** Uncorrected *p* distances of the cyt *b* alignment, based on 1,143 aligned sites.

| | | 30 | 31 | 32 | 33 | 34 | 35 | 36 | 37 | 38 | 39 | 40 | 41 | 42 | 43 | 44 | 45 | 46 | 47 | 48 | 49 | 50 | 51 | 52 |
|---|---|---|---|---|---|---|---|---|---|---|---|---|---|---|---|---|---|---|---|---|---|---|---|---|
| 1 | MTD17171 Gopherus berlandieri | | | | | | | | | | | | | | | | | | | | | | | |
| 2 | MTD18707 Aldabrachelys gigantea | | | | | | | | | | | | | | | | | | | | | | | |
| 3 | AF371241 Aldabrachelys gigantea | | | | | | | | | | | | | | | | | | | | | | | |
| 4 | AF371242 Aldabrachelys gigantea | | | | | | | | | | | | | | | | | | | | | | | |
| 5 | KT613185 Aldabrachelys gigantea | | | | | | | | | | | | | | | | | | | | | | | |
| 6 | AF371240 Aldabrachelys grandidieri | | | | | | | | | | | | | | | | | | | | | | | |
| 7 | MTD18660 Astrochelys radiata | | | | | | | | | | | | | | | | | | | | | | | |
| 8 | AF020897 Astrochelys radiata | | | | | | | | | | | | | | | | | | | | | | | |
| 9 | AF371239 Astrochelys radiata | | | | | | | | | | | | | | | | | | | | | | | |
| 10 | MTD15998 Astrochelys yniphora | | | | | | | | | | | | | | | | | | | | | | | |
| 11 | AF020896 Astrochelys yniphora | | | | | | | | | | | | | | | | | | | | | | | |
| 12 | LT599482 Chelonoidis alburyorum | | | | | | | | | | | | | | | | | | | | | | | |
| 13 | LT599483 Chelonoidis carbonarius | | | | | | | | | | | | | | | | | | | | | | | |
| 14 | LT599484 Chelonoidis chilensis | | | | | | | | | | | | | | | | | | | | | | | |
| 15 | LT599485 Chelonoidis denticulatus | | | | | | | | | | | | | | | | | | | | | | | |
| 16 | AF192932 Chelonoidis abingdonii | | | | | | | | | | | | | | | | | | | | | | | |
| 17 | JN637211 Chelonoidis becki | | | | | | | | | | | | | | | | | | | | | | | |
| 18 | AF192931 Chelonoidis chathamensis | | | | | | | | | | | | | | | | | | | | | | | |
| 19 | AF192940 Chelonoidis darwini | | | | | | | | | | | | | | | | | | | | | | | |
| 20 | AY097816 Chelonoidis donfaustoi | | | | | | | | | | | | | | | | | | | | | | | |
| 21 | MG912820 Chelonoidis duncanensis | | | | | | | | | | | | | | | | | | | | | | | |
| 22 | JN637180 Chelonoidis ephippium | | | | | | | | | | | | | | | | | | | | | | | |
| 23 | AF192933 Chelonoidis hoodensis | | | | | | | | | | | | | | | | | | | | | | | |
| 24 | AF192938 Chelonoidis microphyes | | | | | | | | | | | | | | | | | | | | | | | |
| 25 | JN637231 Chelonoidis niger complex | | | | | | | | | | | | | | | | | | | | | | | |
| 26 | JN999704 Chelonoidis niger complex | | | | | | | | | | | | | | | | | | | | | | | |
| 27 | JN637228 Chelonoidis phantasticus | | | | | | | | | | | | | | | | | | | | | | | |
| 28 | JN637214 Chelonoidis porteri | | | | | | | | | | | | | | | | | | | | | | | |
| 29 | LT599486 Chelonoidis vicina | | | | | | | | | | | | | | | | | | | | | | | |
| 30 | MTD18661 Pyxis arachnoides | — | | | | | | | | | | | | | | | | | | | | | | |
| 31 | AF020894 Pyxis arachnoides | 0.000 | — | | | | | | | | | | | | | | | | | | | | | |
| 32 | MTD1244 Pyxis planicauda | 0.095 | 0.080 | — | | | | | | | | | | | | | | | | | | | | |
| 33 | AF020895 Pyxis planicauda | 0.080 | 0.080 | 0.000 | — | | | | | | | | | | | | | | | | | | | |
| 34 | NHM(UK) 2000.47 Cylindraspis indica | 0.117 | 0.109 | 0.136 | 0.122 | — | | | | | | | | | | | | | | | | | | |
| 35 | NHM(UK) 2000.48 Cylindraspis indica | 0.119 | 0.106 | 0.137 | 0.119 | 0.007 | — | | | | | | | | | | | | | | | | | |
| 36 | NHM(UK) 2000.49 Cylindraspis indica | 0.117 | 0.109 | 0.137 | 0.127 | 0.003 | 0.007 | — | | | | | | | | | | | | | | | | |
| 37 | AF371243 Cylindraspis indica | 0.109 | 0.106 | 0.133 | 0.124 | 0.002 | 0.010 | 0.002 | — | | | | | | | | | | | | | | | |
| 38 | AF371244 Cylindraspis indica | 0.110 | 0.106 | 0.135 | 0.124 | 0.002 | 0.010 | 0.002 | 0.000 | — | | | | | | | | | | | | | | |
| 39 | NHM(UK) R4021 Cylindraspis inepta | 0.119 | 0.104 | 0.136 | 0.117 | 0.017 | 0.019 | 0.017 | 0.017 | 0.017 | — | | | | | | | | | | | | | |
| 40 | NHM(UK) 2000.54 Cylindraspis inepta | 0.117 | 0.104 | 0.132 | 0.111 | 0.016 | 0.019 | 0.016 | 0.017 | 0.017 | 0.003 | — | | | | | | | | | | | | |
| 41 | NHM(UK) 2000.55 Cylindraspis inepta | 0.117 | 0.104 | 0.132 | 0.111 | 0.016 | 0.019 | 0.016 | 0.017 | 0.017 | 0.003 | 0.000 | — | | | | | | | | | | | |
| 42 | NHM(UK) R3991 Cylindraspis triserrata | 0.123 | 0.106 | 0.129 | 0.104 | 0.083 | 0.081 | 0.083 | 0.072 | 0.072 | 0.086 | 0.084 | 0.084 | — | | | | | | | | | | |
| 43 | NHM(UK) R3992 Cylindraspis triserrata | 0.122 | 0.106 | 0.128 | 0.104 | 0.082 | 0.080 | 0.082 | 0.072 | 0.072 | 0.085 | 0.083 | 0.083 | 0.005 | — | | | | | | | | | |
| 44 | AF371248 Cylindraspis triserrata | 0.114 | 0.106 | 0.109 | 0.104 | 0.074 | 0.062 | 0.069 | 0.072 | 0.072 | 0.074 | 0.069 | 0.069 | 0.000 | 0.000 | — | | | | | | | | |
| 45 | NHM(UK) 2000.52 Cylindraspis peltastes | 0.118 | 0.116 | 0.133 | 0.119 | 0.067 | 0.070 | 0.069 | 0.044 | 0.044 | 0.068 | 0.068 | 0.068 | 0.095 | 0.093 | 0.088 | — | | | | | | | |
| 46 | NHM(UK) 2000.53 Cylindraspis peltastes | 0.117 | 0.117 | 0.137 | 0.130 | 0.067 | 0.071 | 0.069 | 0.047 | 0.047 | 0.073 | 0.072 | 0.072 | 0.099 | 0.097 | 0.086 | 0.000 | — | | | | | | |
| 47 | AF371253 Cylindraspis peltastes | 0.116 | 0.117 | 0.136 | 0.130 | 0.044 | 0.047 | 0.049 | 0.047 | 0.047 | 0.054 | 0.054 | 0.054 | 0.086 | 0.086 | 0.086 | 0.000 | 0.000 | — | | | | | |
| 48 | AF371254 Cylindraspis peltastes | 0.116 | 0.117 | 0.136 | 0.130 | 0.044 | 0.047 | 0.049 | 0.047 | 0.047 | 0.054 | 0.054 | 0.054 | 0.086 | 0.086 | 0.086 | 0.000 | 0.000 | 0.000 | — | | | | |
| 49 | NMW1461 Cylindraspis vosmaeri | 0.115 | 0.117 | 0.129 | 0.127 | 0.057 | 0.060 | 0.059 | 0.049 | 0.050 | 0.063 | 0.061 | 0.061 | 0.087 | 0.086 | 0.081 | 0.025 | 0.024 | 0.022 | 0.022 | — | | | |
| 50 | AF371257 Cylindraspis vosmaeri | 0.115 | 0.114 | 0.140 | 0.132 | 0.050 | 0.052 | 0.055 | 0.052 | 0.052 | 0.060 | 0.060 | 0.060 | 0.085 | 0.085 | 0.085 | 0.017 | 0.020 | 0.020 | 0.020 | 0.007 | — | | |
| 51 | AF371259 Cylindraspis vosmaeri | 0.114 | 0.114 | 0.138 | 0.132 | 0.049 | 0.052 | 0.054 | 0.052 | 0.052 | 0.059 | 0.059 | 0.059 | 0.084 | 0.084 | 0.084 | 0.016 | 0.020 | 0.020 | 0.020 | 0.007 | 0.000 | — | |
| 52 | AF371260 Cylindraspis vosmaeri | 0.114 | 0.114 | 0.138 | 0.132 | 0.049 | 0.052 | 0.054 | 0.052 | 0.052 | 0.059 | 0.059 | 0.059 | 0.084 | 0.084 | 0.084 | 0.016 | 0.020 | 0.020 | 0.020 | 0.007 | 0.000 | 0.000 | — |

**Figure S8.** *Continued.*

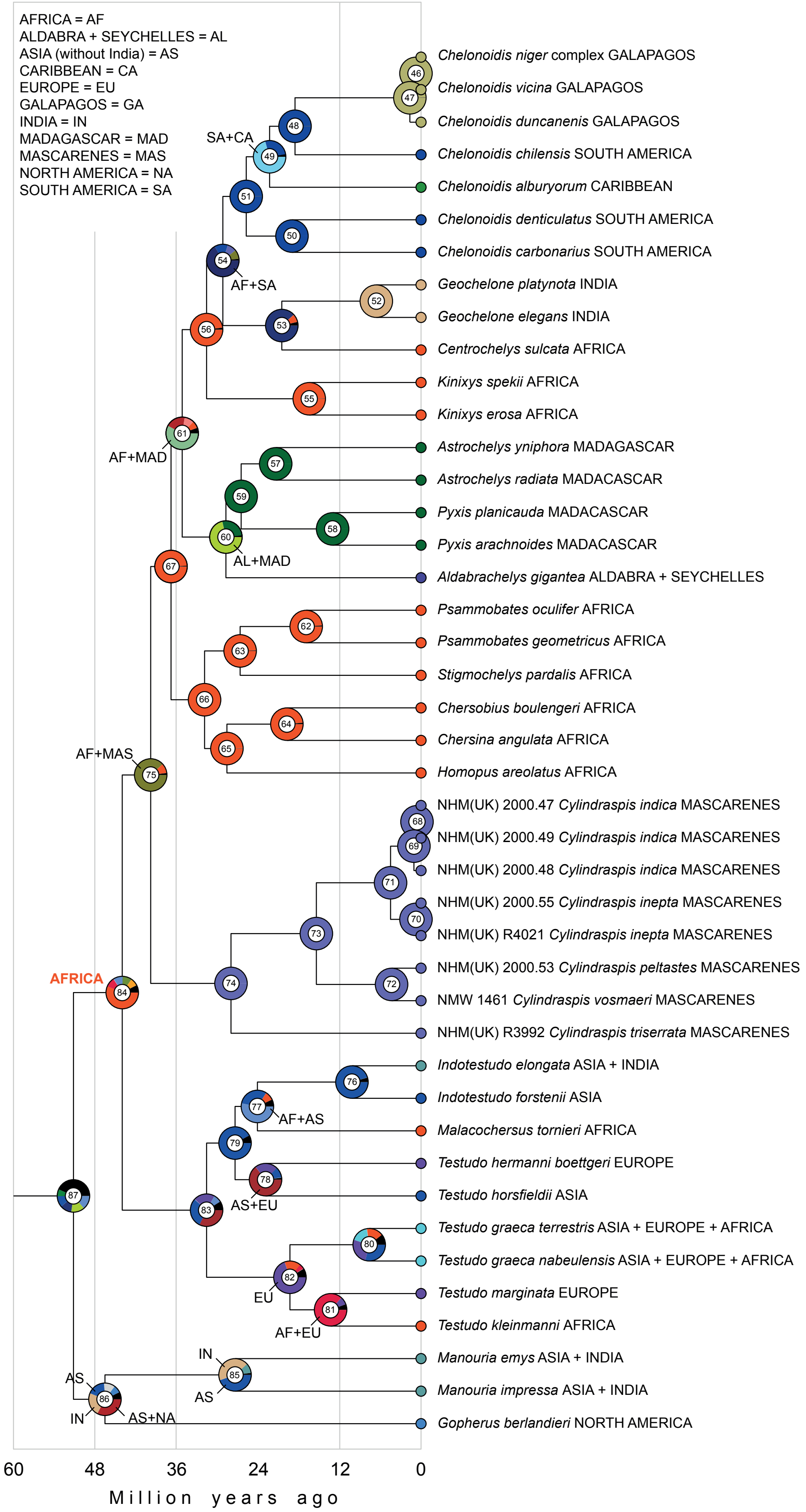


**Figure S9.** Ancestral area reconstruction for tortoises (Testudinidae) using the DIVALIKE model implemented in BioGeoBEARS. The estimated ancestral ranges with the highest ML probability are indicated for each node. Areas with $p < 5\%$ shown in black. Exact percentages are available through Dryad.

**Table S1.** Material of *Cylindraspis* analysed in the present study by NGS methods and by Austin & Arnold (2001) by Sanger sequencing.

| Taxon | Collection and voucher | Island | Locality | Condition | Sample | This study | Austin & Arnold (2001) |
|---|---|---|---|---|---|---|---|
| *C. indica* | MNHN 7819; type | La Réunion | ? | carapace | dried tissue and bone | n/o | AF371243 |
| *C. indica* | MNHN 9374 | La Réunion | ? | carapace | dried tissue and bone | n/o | AF371244 |
| *C. indica* | NHM(UK) 2000.47 | La Réunion | Marais de l'Ermitage | humerus | bone | mt-genome | AF371245 |
| *C. indica* | NHM(UK) 2000.48 | La Réunion | Marais de l'Ermitage | humerus | bone | mt-genome | AF371246 |
| *C. indica* | NHM(UK) 2000.49 | La Réunion | Marais de l'Ermitage | humerus | bone | mt-genome | AF371247 |
| *C. inepta* | NHM(UK) 1876.11.4.17 | Mauritius | Flic en Flaq | femur | bone | low cov. | no PCR |
| *C. inepta* | NHM(UK) 1977.549 | Mauritius | Mare aux Songes | skull | bone | low cov. | no PCR |
| *C. inepta* | NHM(UK) 1977.558 | Mauritius | Mare aux Songes | femur | bone | low cov. | no PCR |
| *C. inepta* | NHM(UK) 1977.572 | Mauritius | Mare aux Songes | skull | bone | low cov. | no PCR |
| *C. inepta* | NHM(UK) 1977.663 | Mauritius | Mare aux Songes | femur | bone | low cov. | no PCR |
| *C. inepta* | NHM(UK) 2000.54 | Mauritius | Ile aux Aigrettes | vertebra | bone | cyt *b* only | AF371252 |
| *C. inepta* | NHM(UK) 2000.55 | Mauritius | Ile aux Aigrettes | vertebra | bone | mt-genome | AF371251 |
| *C. inepta* | NHM(UK) R4021 | Mauritius | Mare aux Songes | femur | bone | mt-genome | AF371250 |
| *C. inepta* | NHM(UK) R4661 | Mauritius | Mare aux Songes | shoulder girdle | bone | n/o | no PCR |
| *C. peltastes* | MNHN 7831, type | Rodrigues | ? | carapace | dried tissue and bone | n/o | AF371253 |
| *C. peltastes* | NHM(UK) 2000.51 | Rodrigues | Caverne Patate | fragment of carapace | bone | low cov. | AF371254 |
| *C. peltastes* | NHM(UK) 2000.52 | Rodrigues | Caverne Bambara | fragment of plastron | bone | cyt *b* only | AF371256 |
| *C. peltastes* | NHM(UK) 2000.53 | Rodrigues | Caverne Bambara | fragment of plastron | bone | mt-genome | AF371255 |
| *C. triserrata* | NHM(UK) 1947.3.5.5 | Mauritius | ? | shell | dried tissue | n/o | AF371248 |
| *C. triserrata* | NHM(UK) 1977.569 | Mauritius | Mare aux Songes | skull | bone | low cov. | no PCR |
| *C. triserrata* | NHM(UK) 1977.661 | Mauritius | Mare aux Songes | femur | bone | low cov. | no PCR |
| *C. triserrata** | NHM(UK) R3991 | Mauritius | Mare aux Songes | femur | bone | cyt *b* only | no PCR |
| *C. triserrata* | NHM(UK) R3992 | Mauritius | Mare aux Songes | femur | bone | mt-genome | AF371249 |
| *C. vosmaeri* | MNHN 1883.558 | Rodrigues | ? | stuffed specimen | dried skin | n/o | AF371259 |
| *C. vosmaeri* | NHM(UK) 2000.50 | Rodrigues | Caverne Patate | fragment of plastron | bone | n/o | AF371260 |
| *C. vosmaeri* | NMW 1461 | Rodrigues | ? | carapace | dried tissue | mt-genome | AF371258 |
| *C. vosmaeri* | RMNH 6001; type | Rodrigues | ? | carapace | dried tissue | n/o | AF371257 |
| *Cylindraspis* sp. | NHM(UK) 1977.641 | Mauritius | Mare aux Songes | pelvis | bone | n/o | no PCR |
| *Cylindraspis* sp. | NHM(UK) R4687 | Mauritius | Mare aux Songes | femur | bone | n/o | no PCR |
| *Cylindraspis* sp. | — | Mauritius | Mount Zaco | fragment of carapace | bone | n/o | no PCR |
| *Cylindraspis* sp. | — | Mauritius | La Prairie | fragment of carapace | bone | n/o | no PCR |

Abbreviations: MNHN = Muséum National d'Histoire naturelle, Paris; NHM(UK) = Natural History Museum of the United Kingdom, London; NMW = Natural History Museum, Vienna; RMNH = Naturalis, Leiden; n/o: sample not obtained for study; low cov.: assembly too fragmentary and with very low coverage; no PCR: no successful PCR amplification.
*Misidentified as *C. inepta* by Austin & Arnold (2001).

**Table S2.** Origin of fresh material and assembly details for mitochondrial genomes generated by amplicon sequencing.

| Taxon | Sample | Origin | Raw reads | Clean reads | Readpool for assembly | Assembled reads | % of readpool | Max. coverage | Average coverage | Average consensus quality (best = 90) | Ambiguous positions in contig |
|---|---|---|---|---|---|---|---|---|---|---|---|
| *Aldabrachelys gigantea* | MTD 18707 | Zoo Dresden | 587,202 | 377,154 | 20,000 | 17,185 | 85.9% | 199 | 105 | 90 | 0% |
| *Astrochelys radiata* | MTD 18660 | Schönbrunn Zoo, Vienna | 1,704,795 | 904,611 | 20,000 | 18,330 | 91.7% | 242 | 116 | 90 | 0% |
| *Astrochelys yniphora* | MTD 15998 | Rodrigues, breeding colony | 396,279 | 281,184 | 20,000 | 19,071 | 95.4% | 204 | 120 | 90 | 0% |
| *Chersina angulata* | MTD 13772 | South Africa, Western Cape, Nuwerus | 1,338,921 | 865,835 | 20,000 | 14,397 | 72% | 152 | 90 | 90 | 0% |
| *Chersobius boulengeri* | MTD 15558 | South Africa, Northern Cape, Victoria West | 1,406,524 | 907,787 | 20,000 | 17,015 | 85.1% | 163 | 107 | 90 | 0% |
| *Geochelone elegans* | MTD 6057 | Sri Lanka | 1,808,781 | 946,603 | 20,000 | 19,097 | 95.5% | 232 | 118 | 90 | 0% |
| *Geochelone platynota* | MTD 4059 | Myanmar | 1,427,411 | 611,064 | 20,000 | 17,817 | 89.1% | 278 | 110 | 90 | 0% |
| *Gopherus berlandieri* | MTD 17171 | Zoo Leipzig | 1,518,677 | 861,702 | 20,000 | 18,762 | 93.8% | 266 | 110 | 84 | 0% |
| *Homopus areolatus* | MTD 15479 | South Africa, Western Cape, Ezelfontein | 1,347,680 | 886,442 | 20,000 | 14,371 | 71.9% | 192 | 90 | 90 | 0% |
| *Kinixys erosa* | MTD 15816 | Dem. Rep. Congo, Equateur, Mongala, Umangi | 1,479,679 | 945,509 | 20,000 | 18,140 | 90.7% | 234 | 112 | 90 | 0% |
| *Kinixys spekii* | MTD 17037 | South Africa, Limpopo, Hoedspruit | 1,336,548 | 842,047 | 20,000 | 13,919 | 69.6% | 236 | 90 | 90 | 0% |
| *Psammobates geometricus* | MTD 13895 | South Africa, Western Cape | 1,643,399 | 824,878 | 20,000 | 16,034 | 80.2% | 205 | 102 | 90 | 0% |
| *Psammobates oculifer* | MTD 18196 | Namibia, Hardap, Mariental | 1,500,345 | 823,479 | 20,000 | 19,115 | 95.6% | 270 | 122 | 90 | 0% |
| *Pyxis arachnoides* | MTD 18661 | Schönbrunn Zoo, Vienna | 1,649,654 | 870,982 | 20,000 | 19,265 | 96.3% | 265 | 125 | 88 | 0% |
| *Pyxis planicauda* | MTD 1244 | Schönbrunn Zoo, Vienna | 1,619,922 | 889,369 | 20,000 | 18,236 | 91.2% | 250 | 117 | 90 | 0% |
| *Stigmochelys pardalis* | MTD 16076 | Captive bred | 540,692 | 243,929 | 20,000 | 19,266 | 96.3% | 237 | 119 | 90 | 0% |

Abbreviation: MTD = Museum of Zoology, Senckenberg Dresden.

**Table S3.** DNA extraction details for *Cylindraspis* samples.

| Taxon | Voucher | Sample | Total sample weight (dry) | Bone powder into extraction | Bone powder out of extraction | DNA conc. (ng/µl) | DNA into ssLib (ng) | Generated data | Extraction protocol |
|---|---|---|---|---|---|---|---|---|---|
| *C. indica* | NHM(UK) 2000.47 | bone | 420.0 mg | 90.0 mg (wet) | 14.0 mg (wet) | 3.2 | 13.0 | mt-genome | Dabney *et al.* (2013) |
| *C. indica* | NHM(UK) 2000.48 | bone | 157.0 mg | 89.0 mg (wet) | 16.0 mg (wet) | 0.4 | 7.2 | mt-genome | Dabney *et al.* (2013) |
| *C. indica* | NHM(UK) 2000.49 | bone | 225.0 mg | 91.0 mg (wet) | 44.0 mg (wet) | 1.1 | 13.0 | mt-genome | Dabney *et al.* (2013) |
| *C. inepta* | NHM(UK) R4021 | bone | 164.0 mg | 73.0 mg (wet) | 13.5 mg (wet) | 1.2 | 13.0 | mt-genome | Dabney *et al.* (2013) |
| | | | | | | | | | |
| *C. inepta* | NHM(UK) 1977.549 | bone | 57.5 mg | 62.0 mg (wet) | 59.0 mg (wet) | 15.3 | 13.8 | low cov. | Dabney *et al.* (2013) |
| *C. inepta* | NHM(UK) 1977.572 | bone | 89.0 mg | 109.0 mg (wet) | 46.5 mg (wet) | 1.4 | 13.0 | low cov. | Dabney *et al.* (2013) |
| *C. inepta* | NHM(UK) 1977.558 | bone | 118.0 mg | 100.0 mg (wet) | 36.0 mg (wet) | 1.0 | 13.0 | low cov. | Dabney *et al.* (2013) |
| *C. inepta* | NHM(UK) 1977.663 | bone | 247.0 mg | 101.0 mg (wet) | 31.0 mg (wet) | 0.2 | 4.8 | low cov. | Dabney *et al.* (2013) |
| *C. inepta* | NHM(UK) 1876.11.4.17 | bone | 158.0 mg | 98.0 mg (wet) | 41.5 mg (wet) | 0.6 | 12.0 | low cov. | Dabney *et al.* (2013) |
| *C. inepta* | NHM(UK) 2000.54 | bone | 60.0 mg | 91.0 mg (wet) | 24.5 mg (wet) | 6.3 | 13.2 | cyt *b* | Dabney *et al.* (2013) |
| *C. inepta* | NHM(UK) 2000.55 | bone | 74.0 mg | 97.0 mg (wet) | 21.0 mg (wet) | 20.6 | 14.4 | mt-genome | Dabney *et al.* (2013) |
| *C. peltastes* | NHM(UK) 2000.51 | bone | 124.0 mg | 80.0 mg (wet) | 11.0 mg (wet) | 6.8 | 13.0 | low cov. | Dabney *et al.* (2013) |
| *C. peltastes* | NHM(UK) 2000.52 | bone | 100.0 mg | 74.0 mg (wet) | 8.0 mg (wet) | 4.1 | 13.2 | cyt *b* | Dabney *et al.* (2013) |
| *C. peltastes* | NHM(UK) 2000.53 | bone | 82.0 mg | 69.0 mg (wet) | 12.0 mg (wet) | 3.8 | 12.9 | mt-genome | Dabney *et al.* (2013) |
| *C. triserrata** | NHM(UK) R3991 | bone | 52.0 mg | 88.0 mg (wet) | 10.0 mg (wet) | 2.9 | 13.2 | cyt *b* | Dabney *et al.* (2013) |
| *C. triserrata* | NHM(UK) R3992 | bone | 96.0 mg | 58.0 mg (dry) | 18.0 mg (wet) | 2.8 | 13.0 | mt-genome | Dabney *et al.* (2013) |
| *C. triserrata* | NHM(UK) 1977.569 | bone | 306.0 mg | 85.0 mg (wet) | 21.0 mg (wet) | 2.9 | 13.0 | low cov. | Dabney *et al.* (2013) |
| *C. triserrata* | NHM(UK) 1977.661 | bone | 261.0 mg | 82.0 mg (wet) | 22.0 mg (wet) | 0.6 | 10.5 | low cov. | Dabney *et al.* (2013) |
| *C. vosmaeri* | NMW 1461 | tissue | — | — | — | 2.2 | 44.4 | mt-genome | Qiagen Blood and Tissue Kit |

Abbreviations: NHM(UK) = Natural History Museum of the United Kingdom, London; NMW = Natural History Museum, Vienna; low cov.: assembly too fragmentary and with very low coverage.
*Misidentified as *C. inepta* by Austin & Arnold (2001).

**Table S4.** Mitochondrial sequences used as starting references for MITObim mapping.

| **Taxon** | **Voucher** | **Reference** |
|---|---|---|
| *Cylindraspis indica* | NHM(UK) 2000.47 | NMW 1461 |
| *Cylindraspis indica* | NHM(UK) 2000.48 | NMW 1461 |
| *Cylindraspis indica* | NHM(UK) 2000.49 | NMW 1461 |
| *Cylindraspis inepta* | NHM(UK) R4021 | NMW 1461 |
| *Cylindraspis inepta* | NHM(UK) 2000.54 | NMW 1461 |
| *Cylindraspis inepta* | NHM(UK) 2000.55 | NMW 1461 |
| *Cylindraspis peltastes* | NHM(UK) 2000.52 | NMW 1461 |
| *Cylindraspis peltastes* | NHM(UK) 2000.53 | NMW 1461 |
| *Cylindraspis triserrata** | NHM(UK) R3991 | NMW 1461 |
| *Cylindraspis triserrata* | NHM(UK) R3992 | NMW 1461 |
| *Cylindraspis vosmaeri* | NMW 1461 | LT599486 |
| Amplicon sequencing: | | |
| *Aldabrachelys gigantea* | MTD 18707 | LT599487 |
| *Astrochelys radiata* | MTD 18660 | LT599487 |
| *Astrochelys yniphora* | MTD 15998 | MTD 18660 |
| *Chersina angulata* | MTD 13772 | LT599487 |
| *Chersobius boulengeri* | MTD 15558 | LT599487 |
| *Geochelone elegans* | MTD 6057 | LT599487 |
| *Geochelone platynota* | MTD 4059 | LT599487 |
| *Gopherus berlandieri* | MTD 17171 | DQ080040 |
| *Homopus areolatus* | MTD 15479 | MTD 15558 |
| *Kinixys erosa* | MTD 15816 | LT599487 |
| *Kinixys spekii* | MTD 17037 | LT599487 |
| *Psammobates geometricus* | MTD 13895 | LT599487 |
| *Psammobates oculifer* | MTD 18196 | LT599487 |
| *Pyxis arachnoides* | MTD 18661 | LT599487 |
| *Pyxis planicauda* | MTD 1244 | LT599487 |
| *Stigmochelys pardalis* | MTD 16076 | LT599487 |

Abbreviations: MTD = Museum of Zoology, Senckenberg Dresden;
NHM(UK) = Natural History Museum of the United Kingdom, London;
NMW = Natural History Museum, Vienna.
*Misidentified as *C. inepta* by Austin & Arnold (2001).

**Table S5.** Individual sequence lengths of *Cylindraspis* material used for phylogenetic analyses.

| Taxon | Voucher | Length of GenBank/ENA sequence | Ambiguous positions | Aligned length for calculations | Ambiguous positions | Source | Accession number |
|---|---|---|---|---|---|---|---|
| *C. indica* | MNHN 7819 | 426 bp | 0 | 405 bp cyt *b* | 0 | Austin & Arnold (2001) | AF371243 |
| *C. indica* | MNHN 9374 | 422 bp | 0 | 401 bp cyt *b* | 0 | Austin & Arnold (2001) | AF371244 |
| *C. indica* | NHM(UK) 2000.47 | 15,345 bp | 0 | 15,234 bp mitogenome | 0 | This study | LR697059 |
| *C. indica* | NHM(UK) 2000.48 | 15,344 bp | 0 | 15,234 bp mitogenome | 0 | This study | LR697060 |
| *C. indica* | NHM(UK) 2000.49 | 15,345 bp | 15 | 15,234 bp mitogenome | 15 | This study | LR697061 |
| *C. inepta* | NHM(UK) 2000.54 | 1,144 bp | 0 | 1,143 bp cyt *b* | 0 | This study | LR694548 |
| *C. inepta* | NHM(UK) 2000.55 | 15,348 bp | 2 | 15,234 bp mitogenome | 2 | This study | LR697063 |
| *C. inepta* | NHM(UK) R4021 | 15,348 bp | 0 | 15,234 bp mitogenome | 0 | This study | LR697062 |
| *C. peltastes* | NHM(UK) 2000.51 | 426 bp | 0 | 405 bp cyt *b* | 0 | Austin & Arnold (2001) | AF371254 |
| *C. peltastes* | NHM(UK) 2000.52 | 1,144 bp | 170 | 1,143 bp cyt *b* | 170 | This study | LR694549 |
| *C. peltastes* | NHM(UK) 2000.53 | 15,346 bp | 0 | 15,234 bp mitogenome | 0 | This study | LR697064 |
| *C. peltastes* | MNHN 7831 | 426 bp | 0 | 405 bp cyt *b* | 0 | Austin & Arnold (2001) | AF371253 |
| *C. triserrata* | NHM(UK) 1947.3.5.5 | 425 bp | 0 | 405 bp cyt *b* | 0 | Austin & Arnold (2001) | AF371248 |
| *C. triserrata** | NHM(UK) R3991 | 1,144 bp | 0 | 1,143 bp cyt *b* | 0 | This study | LR694550 |
| *C. triserrata* | NHM(UK) R3992 | 15,335 bp | 2 | 15,232 bp mitogenome | 2 | This study | LR697065 |
| *C. vosmaeri* | MNHN 1883.558 | 426 bp | 0 | 405 bp cyt *b* | 0 | Austin & Arnold (2001) | AF371259 |
| *C. vosmaeri* | NHM(UK) 2000.50 | 426 bp | 0 | 405 bp cyt *b* | 0 | Austin & Arnold (2001) | AF371260 |
| *C. vosmaeri* | NMW 1461 | 15,344 bp | 0 | 15,234 bp mitogenome | 0 | This study | LR697066 |
| *C. vosmaeri* | RMNH 6001 | 422 bp | 0 | 401 bp cyt *b* | 0 | Austin & Arnold (2001) | AF371257 |

Abbreviations: MNHN = Muséum National d'Histoire naturelle, Paris; NHM(UK) = Natural History Museum of the United Kingdom, London; NMW = Natural History Museum, Vienna; RMNH = Naturalis, Leiden.
*Misidentified as *C. inepta* by Austin & Arnold (2001).

**Table S6.** PCR primer for amplicon sequencing of fresh material and bait-library preparation.

| **Long-range PCR primer pairs designed for amplicon sequencing and bait-library preparation** | | **Source** |
|---|---|---|
| LR1 For Baits | 5´-RTGGCAYTGAAGHTGYCRAGATG-3´ | this study |
| LR1 Rev Baits | 5´-TGRATTRTRGCTACTGCYAGYTC-3´ | this study |
| LR2 For Baits | 5´-CTYACAGCMAAYYTAACAGCYGG-3´ | this study |
| LR2 For Centrochelys | 5´-CTCACAGCTAATCTAACAGCTGG-3´ | this study |
| LR2 Rev Centrochelys | 5´-CTTTGATTGTTAAGCTACTGG-3´ | this study |
| LR1 Rev Chelonoidis | 5´-TGGATTATRGCTACTGCTAGTTC-3´ | this study |
| LR2 For Chelonoidis | 5´-CTWACAGCYAACCTAACAGCTGG-3´ | this study |
| LR2 For Geochelone elegans | 5´-CATTACTATACTACTCACAGATCG-3´ | this study |
| LR2 Rev Geochelone elegans | 5´-ATCTTACTTACAAGGGTTGC-3´ | this study |
| LR2 For Homopus | 5´-TTCTTTGACCCTTCAGGAGG-3´ | this study |
| LR2 For Psammobates | 5´-TTCTTCGACCCTTCAGGAGG-3´ | this study |
| LR1 For Pyxis arachnoides | 5´-ATGCTTAGCCTTAAATCCAG-3´ | this study |
| LR1 Rev Pyxis arachnoides | 5´-TCCGAGTTTTAGTAATACTGC-3´ | this study |
| LR2 For Pyxis arachnoides | 5´-GACCCAATTCTATACCAACACC-3´ | this study |
| LR1 For Stigmochelys | 5´-ATGGCACTGAAGTTGCCAAGATG-3´ | this study |
| LR1 Rev Stigmochelys | 5´-TGGATTATGGCTACTGCTAGTTC-3´ | this study |
| LR2 For Stigmochelys | 5´-CTTACAGCAAATTTAACAGCTGG-3´ | this study |
| mt-f-na v2 | 5´-TCAGTTTTTGGTTTACAAGACC-3´ | this study |
| **Internal Sanger sequencing primers for 12S and cyt *b*** | | |
| 12S-L1091 | 5´-AAAAAGCTTCAAACTGGGATTAGATACCCCACTAT-3´ | Kocher *et al.*(1989) |
| mt-c-For2 | 5´-TGAGGVCARATATCATTYTGAG-3´ | Fritz *et al.* (2006) |

**Table S7.** Long-range PCR conditions for amplicon sequencing and bait library preparation. Provided are the amount of template DNA per PCR reaction, primer combinations, PCR conditions (number of repetitive cycles and annealing temperature), multiple PCR products present and target product cut from agarose gel, length of PCR product. Minimum overlap between LR1 and LR2 of at least 106 bp depending on the primer combinations, e.g., LR1 For Baits/LR1 Rev Baits and LR2 For Baits/mt-f-na v2.

| Taxon | Voucher | DNA | | Forward primer | Reverse primer | Cycles | Annealing temp. | Cut from gel | Length |
|---|---|---|---|---|---|---|---|---|---|
| Amplicon sequencing | | | | | | | | | |
| *Aldabrachelys gigantea* | MTD 18707 | 4.0 ng | LR1 | LR1 For Baits | LR1 Rev Baits | 35x | 50°C | X | ~8,700 bp |
| | | | LR2 | LR2 For Baits | mt-f-na v2 | 35x | 50°C | | ~7,100 bp |
| *Astrochelys radiata* | MTD 18660 | 3.6 ng | LR1 | LR1 For Baits | LR1 Rev Baits | 35x | 50°C | X | ~8,700 bp |
| | | | LR2 | LR2 For Baits | mt-f-na v2 | 35x | 50°C | | ~7,100 bp |
| *Astrochelys yniphora* | MTD 15998 | 6.3 ng | LR1 | LR1 For Baits | LR1 Rev Baits | 35x | 50°C | X | ~8,700 bp |
| | | | LR2 | LR2 For Baits | mt-f-na v2 | 35x | 50°C | | ~7,100 bp |
| *Chersina angulata* | MTD 13772 | 7.6 ng | LR1 | LR1 For Baits | LR1 Rev Baits | 37x | 50°C | | ~8,700 bp |
| | | | LR2 | LR2 For Baits | mt-f-na v2 | 35x | 50°C | | ~7,100 bp |
| *Chersobius boulengeri* | MTD 15558 | 6.9 ng | LR1 | LR1 For Baits | LR1 Rev Baits | 35x | 50°C | | ~8,700 bp |
| | | | LR2 | LR2 For Baits | mt-f-na v2 | 37x | 55°C | | ~7,100 bp |
| *Geochelone elegans* | MTD 6057 | 14.0 ng | LR1 | LR1 For Baits | LR1 Rev Baits | 37x | 50°C | X | ~8,700 bp |
| | | | LR2 | LR2 For Geochelone elegans | LR2 Rev Geochelone elegans | 37x | 50°C | | ~9,730 bp |
| *Geochelone platynota* | MTD 4059 | 23.6 ng | LR1 | LR1 For Baits | LR1 Rev Baits | 35x | 50°C | X | ~8,700 bp |
| | | | LR2 | LR2 For Baits | mt-f-na v2 | 35x | 50°C | | ~7,100 bp |
| *Gopherus berlandieri* | MTD 17171 | 5.8 ng | LR1 | LR1 For Baits | LR1 Rev Baits | 35x | 50°C | X | ~8,700 bp |
| | | | LR2 | LR2 For Baits | mt-f-na v2 | 35x | 50°C | | ~7,100 bp |
| *Homopus areolatus* | MTD 15479 | 9.3 ng | LR1 | LR1 For Baits | LR1 Rev Baits | 35x | 50°C | | ~8,700 bp |
| | | | LR2 | LR2 For Homopus | mt-f-na v2 | 37x | 50°C | | ~8,600 bp |
| *Kinixys erosa* | MTD 15816 | 6.4 ng | LR1 | LR1 For Baits | LR1 Rev Baits | 33x | 50°C | X | ~8,700 bp |
| | | | LR2 | LR2 For Baits | mt-f-na v2 | 40x | 53°C | | ~7,100 bp |
| *Kinixys spekii* | MTD 17037 | 32.0 ng | LR1 | LR1 For Baits | LR1 Rev Baits | 37x | 50°C | X | ~8,700 bp |
| | | | LR2 | LR2 For Baits | mt-f-na v2 | 35x | 55°C | | ~7,100 bp |
| *Psammobates geometricus* | MTD 13895 | 9.3 ng | LR1 | LR1 For Baits | LR1 Rev Baits | 35x | 50°C | | ~8,700 bp |
| | | | LR2 | LR2 For Psammobates | mt-f-na v2 | 35x | 55°C | | ~8,600 bp |

**Table S7.** *Continued*.

| Taxon | Voucher | DNA | | Forward primer | Reverse primer | Cycles | Annealing temp. | Cut from gel | Length |
|---|---|---|---|---|---|---|---|---|---|
| *Psammobates oculifer* | MTD 18196 | 9.6 ng | LR1 | LR1 For Baits | LR1 Rev Baits | 35x | 50°C | X | ~8,700 bp |
| | | | LR2 | LR2 For Baits | mt-f-na v2 | 35x | 50°C | | ~7,100 bp |
| *Pyxis arachnoides* | MTD 18661 | 3.2 ng | LR1 | LR1 For Pyxis arachnoides | LR1 Rev Pyxis arachnoides | 35x | 50°C | | ~10,450 bp |
| | | | LR2 | LR2 For Pyxis arachnoides | mt-f-na v2 | 37x | 50°C | X | ~9,330 bp |
| *Pyxis planicauda* | MTD 1244 | 14.5 ng | LR1 | LR1 For Baits | LR1 Rev Baits | 30x | 55°C | | ~8,700 bp |
| | | | LR2 | LR2 For Baits | mt-f-na v2 | 35x | 50°C | X | ~7,100 bp |
| *Stigmochelys pardalis* | MTD 16076 | 12.4 ng | LR1 | LR1 For Stigmochelys | LR1-Rev Stigmochelys | 35x | 50°C | X | ~8,700 bp |
| | | | LR2 | LR2 For Stigmochelys | mt-f-na v2 | 35x | 50°C | | ~7,100 bp |
| Bait library preparation | | | | | | | | | |
| *Centrochelys sulcata* | MTD 16072 | 7.2 ng | LR1 | LR1 For Baits | LR1 Rev Baits | 37x | 50°C | | ~8,700 bp |
| | | | LR2 | LR2 For Centrochelys | LR2 Rev Centrochelys | 30x | 55°C | | ~7,100 bp |
| *Chelonoidis chilensis* | MTD 5657 | 9.5 ng | LR1 | LR1 For Baits | LR1 Rev Chelonoidis | 30x | 55°C | | ~8,700 bp |
| | | | LR2 | LR2 For Chelonoidis | mt-f-na v2 | 30x | 55°C | | ~7,100 bp |

Abbreviation: MTD = Museum of Zoology, Senckenberg Dresden.

**Table S8.** Mitochondrial genome organisation and annotation of the mitogenome alignment used for analyses, indicating missing regions.

| Positions | Annotation | Strand |
|---|---|---|
| Missing | part of tRNA-Phe | + |
| <1–25 | tRNA-Phe | + |
| 26–1039 | 12S | + |
| 1040–1114 | tRNA-Val | + |
| 1115–2799 | 16S | + |
| 2800–2876 | tRNA-Leu | + |
| 2877–3854 | ND1 | + |
| 3855–3924 | tRNA-Ile | + |
| 3925–3996 | tRNA-Gln | - |
| 3997–4066 | tRNA-Met | + |
| 4067–5104 | ND2 | + |
| 5105–5185 | tRNA-Trp | + |
| 5186–5254 | tRNA-Ala | - |
| 5255–5328 | tRNA-Asn | - |
| 5329–5398 | tRNA-Cys | - |
| 5399–5485 | tRNA-Tyr | - |
| 5486–7024 | COI | + |
| 7025–7087 | tRNA-Ser | - |
| 7088–7159 | tRNA-Asp | + |
| 7160–7846 | COII | + |
| 7847–7924 | tRNA-Lys | + |
| 7925–8095 | atp8 | + |
| 8096–8764 | atp6 | + |
| 8765–9544 | COIII | + |
| 9545–9614 | tRNA-Gly | + |
| 9615–9962 | ND3 | + |
| 9963–10035 | tRNA-Arg | + |
| 10036–10326 | ND4L | + |
| 10327–11700 | ND4 | + |
| 11701–11772 | tRNA-His | + |
| 11773–11841 | tRNA-Ser | + |
| 11842–11914 | tRNA-Leu | + |
| 11915–13732 | ND5 | + |
| 13733–14267 | ND6 | - |
| 14268–14337 | tRNA-Glu | - |
| 14338–15480 | cyt *b* | + |
| 15481–15510> | tRNA-Thr | + |
| missing | part of tRNA-Thr | + |
| missing | tRNA-Pro | presumably - |
| missing | CR | presumably - |

**Table S9.** Evolutionary models for mitogenomes suggested by PartitionFinder2 for RAxML and MrBayes analyses. Best partitioning scheme: partitioned by codon position plus three additional partitions for non-protein-coding DNA (12S, 16S, tRNAs combined).

| Alignment position | Partition | Model RAxML | Model MrBayes |
|---|---|---|---|
| 1–25 | tRNA | GTR+I+G | GTR+I+G |
| 26–1039 | 12S | GTR+I+G | GTR+I+G |
| 1040–1114 | tRNA | GTR+I+G | GTR+I+G |
| 1115–2799 | 16S | GTR+I+G | GTR+I+G |
| 2800–2876 | tRNA | GTR+I+G | GTR+I+G |
| 2877–3854\3 | ND1_pos1 | GTR+I+G | SYM+I+G |
| 2878–3854\3 | ND1_pos2 | GTR+I+G | HKY+I+G |
| 2879–3854\3 | ND1_pos3 | GTR+G | HKY+G |
| 3855–4066 | tRNA | GTR+I+G | GTR+I+G |
| 4067–5104\3 | ND2_pos1 | GTR+I+G | GTR+I+G |
| 4068–5104\3 | ND2_pos2 | GTR+I+G | HKY+I+G |
| 4069–5104\3 | ND2_pos3 | GTR+G | HKY+G |
| 5105–5485 | tRNA | GTR+I+G | GTR+I+G |
| 5486–7024\3 | coxI_pos1 | GTR+I+G | SYM+I+G |
| 5487–7024\3 | coxI_pos2 | GTR+I+G | HKY+I |
| 5488–7024\3 | coxI_pos3 | GTR+I+G | GTR+I+G |
| 7025–7159 | tRNA | GTR+I+G | GTR+I+G |
| 7160–7846\3 | coxII_pos1 | GTR+I+G | K80+I+G |
| 7161–7846\3 | coxII_pos2 | GTR+G | HKY+I+G |
| 7162–7846\3 | coxII_pos3 | GTR+I+G | GTR+I+G |
| 7847–7924 | tRNA | GTR+I+G | GTR+I+G |
| 7925–8095\3 | atp8_pos1 | GTR+G | HKY+G |
| 7926–8095\3 | atp8_pos2 | GTR+G | HKY+G |
| 7927–8095\3 | atp8_pos3 | GTR+I+G | HKY+I+G |
| 8096–8764\3 | atp6_pos1 | GTR+G | GTR+G |
| 8097–8764\3 | atp6_pos2 | GTR+I+G | GTR+I+G |
| 8098–8764\3 | atp6_pos3 | GTR+G | GTR+G |
| 8765–9544\3 | coxIII_pos1 | GTR+I+G | SYM+I+G |
| 8766–9544\3 | coxIII_pos2 | GTR+I+G | HKY+I+G |
| 8767–9544\3 | coxIII_pos3 | GTR+G | GTR+G |
| 9545–9614 | tRNA | GTR+I+G | GTR+I+G |
| 9615–9962\3 | ND3_pos1 | GTR+I+G | K80+I+G |
| 9616–9962\3 | ND3_pos2 | GTR+G | HKY+G |
| 9617–9962\3 | ND3_pos3 | GTR+G | HKY+G |
| 9963–10035 | tRNA | GTR+I+G | GTR+I+G |
| 10036–10326\3 | ND4L_pos1 | GTR+G | HKY+G |
| 10037–10326\3 | ND4L_pos2 | GTR+G | HKY+G |
| 10038–10326\3 | ND4L_pos3 | GTR+I+G | GTR+I+G |
| 10327–11700\3 | ND4_pos1 | GTR+I+G | GTR+I+G |
| 10328–11700\3 | ND4_pos2 | GTR+I+G | GTR+I+G |
| 10329–11700\3 | ND4_pos3 | GTR+G | GTR+G |
| 11701–11914 | tRNA | GTR+I+G | GTR+I+G |
| 11915–13732\3 | ND5_pos1 | GTR+G | GTR+G |
| 11916–13732\3 | ND5_pos2 | GTR+I+G | HKY+I+G |
| 11917–13732\3 | ND5_pos3 | GTR+I+G | GTR+I+G |
| 13733–14267\3 | ND6_pos3 | GTR+G | GTR+G |
| 13734–14267\3 | ND6_pos2 | GTR+G | HKY+G |
| 13735–14267\3 | ND6_pos1 | GTR+I+G | GTR+I+G |
| 14268–14337 | tRNA | GTR+I+G | GTR+I+G |
| 14338–15480\3 | cytb_pos1 | GTR+I+G | GTR+I+G |
| 14339–15480\3 | cytb_pos2 | GTR+I+G | GTR+I+G |
| 14340–15480\3 | cytb_pos3 | GTR+I+G | GTR+I+G |
| 15481–15510 | tRNA | GTR+I+G | GTR+I+G |

**Table S10.** Calibration points used for the molecular dating with BEAST. The implemented priors followed lognormal distributions. Dates were set in million years ago.

| Node | Mean | SD | Offset | 2.5%–97.5% quantiles of the prior distribution | Fossils and references |
|---|---|---|---|---|---|
| Split between Geoemydidae–Testudinidae | 25.4 | 0.5 | 50.3 | 58.7 – 110 | *Hadrianus majusculus* Hay, 1904 |
| *Chelonoidis carbonarius–C. denticulatus* | 10.75 | 0.5 | 11.8 | 15 – 36.8 | *Chelonoidis hesternus* (Auffenberg, 1971) |
| Crown Testudinidae | 16 | 0.5 | 33.9 | 39.2 – 71.5 | *Cheirogaster maurini* Bergounioux, 1935 and *Gigantochersina ammon* (Andrews, 1904) |
| Crown Testudininae | 6 | 0.6 | 33.9 | 35.5 – 50.1 | *Cheirogaster maurini* Bergounioux, 1935 |

**Table S11.** Comparison of the fit of three models in BioGeoBEARS analysis. The best model (DIVALIKE) is highlighted in bold.

| | Ln*L* | Number of parameters | *d* | *e* | AIC | AIC wt |
|---|---|---|---|---|---|---|
| DEC | -102 | 2 | 0.010 | 0.01 | 208.4 | 1.7E-5 |
| **DIVALIKE** | **-99.44** | **2** | **0.007** | **0.005** | **203.2** | **0.0002** |
| BAYAREALIKE | -101.5 | 2 | 0.005 | 0.02 | 207.2 | 3E-5 |

Abbreviations: *d* = dispersal rate; *e* = extinction rate.

**Table S12.** Assembly details of *Cylindraspis* material and blanks generated by shotgun sequencing and hybridization capture.

| Taxon | Voucher | Raw reads | Readpool for assembly | Assembled reads | % of readpool | Average length of assembled reads | Max. coverage | Average coverage | Average consensus quality (best = 90) | Assembled contig length before quality control | Ambiguous positions in contig | Generated |
|---|---|---|---|---|---|---|---|---|---|---|---|---|
| Shotgun sequencing | | | | | | | | | | | | |
| *C. indica* | NHM(UK) 2000.49 | 2,394,268 | 1,720,527 | 351 | 0.02% | | 29 | 5 | 29 | 15,231 | 55.7% | |
| Hybridization capture | | | | | | | | | | | | |
| *C. indica* | NHM(UK) 2000.47 | 2,027,566 | 1,098,706 | 44.477 | 4.05% | 66 bp | 551 | 191 | 90 | 15,563 | 0% | mt-genome |
| *C. indica* | NHM(UK) 2000.48 | 2,027,512 | 1,234,255 | 79.183 | 6.42% | 62 bp | 1012 | 320 | 90 | 15,565 | 0% | mt-genome |
| *C. indica* | NHM(UK) 2000.49 | 2,684,081 | 1,887,317 | 53.433 | 2.83% | 61 bp | 634 | 213 | 89 | 15,571 | 0.1% | mt-genome |
| *C. inepta* | NHM(UK) 2000.54 | 4,408,604 | 1,957,701 | 6.372 | 0.33% | 73 bp | 466 | 33 | 78 | 15,566 | 4.67% | cyt *b* |
| *C. inepta* | NHM(UK) 2000.55 | 3,923,097 | 860,293 | 24.957 | 2.9% | 79 bp | 611 | 129 | 89 | 15,640 | 4.65% | mt-genome |
| *C. inepta* | NHM(UK) R4021 | 1,787,677 | 762,526 | 42.008 | 14% | 67 bp | 521 | 183 | 90 | 15,567 | 0% | mt-genome |
| *C. peltastes* | NHM(UK) 2000.52 | 3,873,302 | 1,154,541 | 7.636 | 0.66% | 68 bp | 634 | 75 | 75 | 15,675 | 5.43% | cyt *b* |
| *C. peltastes* | NHM(UK) 2000.53 | 1,814,251 | 402,648 | 18.519 | 4.6% | 73 bp | 442 | 90 | 89 | 15,584 | 0% | mt-genome |
| *C. triserrata** | NHM(UK) R3991 | 2,908,837 | 1,281,689 | 4.598 | 0.36% | 55 bp | 132 | 19 | 79 | 15,563 | 5.65% | cyt *b* |
| *C. triserrata* | NHM(UK) R3992 | 1,747,986 | 1,070,568 | 11.800 | 1.1% | 63 bp | 256 | 51 | 88 | 15,555 | 0% | mt-genome |
| *C. vosmaeri* | NMW 1461 | 2,260,456 | 1,581,843 | 28.923 | 28.92% | 83 bp | 386 | 157 | 89 | 15,568 | 0% | mt-genome |
| EB1 | | 1,292,905 | 151,304 | 142 | 0.09% | 71 bp | 9 | 4 | 20 | 15,570 | 65.17% | |
| EB2 | | 1,106,659 | 102,262 | 971 | 0.95% | 77 bp | 32 | 8 | 32 | 15,566 | 12.41% | |
| LB1 | | 1,087,937 | 35,862 | 319 | 0.89% | 55 bp | 214 | 4 | 18 | 15,569 | 65.37% | |
| LB2 | | 1,129,390 | 37,805 | 165 | 0.44% | 65 bp | 29 | 4 | 21 | 15,564 | 62.49% | |
| LB3 | | 946,020 | 29,166 | 200 | 0.69% | 68 bp | 15 | 4 | 27 | 15,566 | 54.99% | |

Abbreviations: NHM(UK) = Natural History Museum of the United Kingdom, London; NMW = Natural History Museum, Vienna; EB: extraction blank; LB: library blank.
*Misidentified as *C. inepta* by Austin & Arnold (2001).

## References


Arnold, E. N. & Bour, R. A new *Nactus* gecko (Gekkonidae) and a new *Leiolopisma* skink (Scincidae) from La Réunion, Indian Ocean, based on recent fossil remains and ancient DNA sequence. *Zootaxa* **1705**, 40–50 (2008).

Austin, J. J. & Arnold, E. N. Ancient mitochondrial DNA and morphology elucidate an extinct island radiation of Indian Ocean giant tortoises (*Cylindraspis*). *Proc. R. Soc. B* **268**, 2515–2523 (2001).

Austin, J. J. & Arnold, E. N. Using ancient and recent DNA to explore relationships of extinct and endangered *Leiolopisma* skinks (Reptilia: Scincidae) in the Mascarene islands. *Mol. Phylogenet. Evol*. **39**, 503–511 (2006).

Austin, J. J., Arnold, E. N. & Bour, R. Was there a second adaptive radiation of giant tortoises in the Indian Ocean? Using mitochondrial DNA to investigate speciation and biogeography of *Aldabrachelys* (Reptilia, Testudinidae). *Mol. Ecol*. **12**, 1415–1424 (2003).

Bensasson, D., Zhang, D.-X., Hartl, D. L. & Hewitt, G. M. Mitochondrial pseudogenes: evolution's misplaced witnesses. *Trends Ecol. Evol*. **16**, 314–321 (2001).

Besnard, G. *et al*. Shotgun sequencing of the mitochondrial genome of the Aldabra giant tortoise (*Aldabrachelys gigantea*). *Mitochondrial DNA Part A* **27**, 4543–4544 (2016).

Bour, R., Mourer-Chauviré, C. & Ribes S. Morphology and palaeontological exploration (until 2000) of the extinct tortoises of the Mascarene islands in *Western Indian Ocean Tortoises. Ecology, Diversity, Evolution, Conservation, Palaeontology* (ed Gerlach, J.), 121–202 (Siri Scientific Press, 2014).

Bradler, S., Cliquennois, N. & Buckley, T. R. Single origin of the Mascarene stick insects: ancient radiation on sunken islands? *BMC Evol. Biol.* **15**, 196 (2015).

Brinkman, D. B., Li, J. & Ye, X. Order Testudines in *The Chinese Fossil Reptiles and Their Kin, Second Edition* (eds Li, J., Wu, X. & Zhang, F.), 35–102 (Science Press, 2008).

Crumly, C. R. A hypothesis for the relationships of land tortoise genera (family Testudinidae). *Stud. Geol. Salamant. Vol. Esp*. **1**, 115–124 (1985).

Cui, H. *et al*. Comprehensive next-generation sequence analyses of the entire mitochondrial genome reveal new insights into the molecular diagnosis of mitochondrial DNA disorders. *Genet. Med*. **15**, 338–394 (2013).

Dabney, J. *et al*. Complete mitochondrial genome sequence of a Middle Pleistocene cave bear reconstructed from ultrashort DNA fragments. *Proc. Natl Acad. Sci. USA* **110**, 15758–15763 (2013).

Danilov, I. G. Die fossilen Schildkröten Europas in *Handbuch der Reptilien und Amphibien Europas. Band 3/IIIB: Schildkröten (Testudines) II* (ed Fritz, U.), 329–441 (Aula-Verlag, 2005).

de la Fuente, M. S., Zacarías, G. G. & Vlachos, E. A review of the fossil record of South American turtles of the clade Testudinoidea. *Bull. Peabody Mus. Nat. Hist*. **59**, 269–286 (2018).

de Lapparent de Broin, F. The European turtle fauna from the Triassic to the Present. *Dumerilia* **4**, 155–217 (2001).

Drummond, A. J., Suchard, M. A., Xie, D. & Rambaut, A. Bayesian phylogenetics with BEAUti and the BEAST 1.7. *Mol. Biol. Evol*. **29**, 1969–1973 (2012).

Duncan, R. A., Backman, J. & Peterson, L. Réunion hotspot activity through Tertiary time: initial results from the Ocean Drilling Program, Leg 115. *J. Volcanol. Geoth. Res.* **36**, 193–198 (1989).

Duncan, R. A. & Hargraves, R. B. $^{40}$Ar/$^{39}$Ar geochronology of basement rocks from the Mascarene Plateau, the Chagos Bank, and the Maldives Ridge. *Proc. Ocean Drill. Prog. Sci. Results* **115**, 43–51 (1990).

Fritz, U. & Bininda-Emonds, O. R. P. When genes meet nomenclature: tortoise phylogeny and the shifting generic concepts of *Testudo* and *Geochelone*. *Zoology* **110**, 298–307 (2007).

Fritz, U. *et al*. A rangewide phylogeography of Hermann's tortoise, *Testudo hermanni* (Reptilia: Testudines: Testudinidae): implications for taxonomy. *Zool. Scr*. **35**, 531–543 (2006).

Fritz, U. *et al*. Mitochondrial phylogeography and subspecies of the wide-ranging sub-Saharan leopard tortoise *Stigmochelys pardalis* (Testudines: Testudinidae) – a case study for the pitfalls of pseudogenes and GenBank sequences. *J. Zool. Syst. Evol. Res*. **48**, 348–359 (2010).

Fritz, U. *et al*. Molecular phylogeny of Central and South American slider turtles: implications for biogeography and systematics (Testudines: Emydidae: *Trachemys*). *J. Zool. Syst. Evol. Res*. **50**, 125–136 (2012).

Fritz, U., Gemel, R., Kehlmaier, C., Vamberger, M. & Praschag, P. Phylogeography of the Asian softshell turtle *Amyda cartilaginea* (Boddaert, 1770): evidence for a species complex. *Vertebr. Zool*. **64**, 229–243 (2014).

Gadow, H. On the remains of some gigantic land tortoises, and of an extinct lizard recently discovered in Mauritius. *Trans. Linn. Soc. London* (*Zool*.) **13**, 313–324, pls 42–44 (1894).

Galtier, N. Delineating species in the speciation continuum: a proposal. *Evol. Appl*. **12**, 657–663 (2019).

Hawlitschek, O., Ramírez Garrido, S. & Glaw, F. How marine currents influenced the widespread natural overseas dispersal of reptiles in the Western Indian Ocean region. *J. Biogeogr*. **44**, 1435–1440 (2017).

Joyce, W. G., Parham, J. F., Lyson, T. R., Warnock, R. C. M. & Donoghue, P. C. J. A divergence dating analysis of turtles using fossil calibrations: an example of best practices. *J. Paleontol*. **87**, 612–634 (2013).

Joyce, W. G., Rabi, M., Clark, J. M. & Xu, X. A toothed turtle from the Late Jurassic of China and the global biogeographic history of turtles. *BMC Evol. Biol.* **16**, 236 (2016).

Kehlmaier, C. *et al*. Tropical ancient DNA reveals relationships of the extinct Bahamian giant tortoise *Chelonoidis alburyorum*. *Proc. R. Soc. B* **284**, 20162235 (2017).

Kocher, T. D. *et al*. Dynamics of mitochondrial DNA evolution in mammals: amplification and sequencing with conserved primers. *Proc. Natl Acad. Sci. USA* **86**, 6196–6200 (1989).

Kumar, S., Stecher, G. & Tamura, K. MEGA7: Molecular Evolutionary Genetics Analysis Version 7.0 for bigger datasets. *Mol. Biol. Evol*. **33**, 1870–1874 (2016).

Lanfear, R., Frandsen, P. B., Wright, A. M., Senfeld, T. & Calcott, B. PartitionFinder2: new methods for selecting partitioned models of evolution for molecular and morphological phylogenetic analyses. *Mol. Biol. Evol*. **34**, 772–773 (2016).

Le, M. & Raxworthy, C. J. Human-mediated dispersals do not explain tortoise distribution on the Indian Ocean's islands. *J. Biogeogr*. **44**, 2421–2424 (2017).

Le, M., Raxworthy, C. J., McCord, W. P. & Mertz, L. A molecular phylogeny of tortoises (Testudines: Testudinidae) based on mitochondrial and nuclear genes. *Mol. Phylogenet. Evol*. **40**, 517–531 (2006).

Loire, E. & Galtier, N. Lacking conservation genomics in the giant Galápagos tortoise. *bioRxiv* **101980** (2017).

Lourenço, J. M., Glémin, S., Chiari, Y. & Galtier, N. The determinants of the molecular substitution process in turtles. *J. Evol. Biol*. **26**, 38–50 (2011).

Matzke, N. J. Probabilistic historical biogeography: new models for founder-event speciation, imperfect detection, and fossils allow improved accuracy and model-testing. *Front. Biogeogr*. **5**, 242–248 (2013).

Matzke, N. J. Model selection in historical biogeography reveals that founder-event speciation is a crucial process in island clades. *Syst. Biol*. **63**, 951–970 (2014).

Parham, J. F. *et al*. The phylogeny of Mediterranean tortoises and their close relatives based on complete mitochondrial genome sequences from museum specimens. *Mol. Phylogenet. Evol*. **38**, 50–64 (2006).

Rambaut, A. & Drummond, A. J. TRACER — MCMC trace analysis tool version v1.5. http://beast.bio.ed.ac.uk (2007).

Rambaut, A., Drummond, A. J., Xie, D., Baele, G. & Suchard, M. A. Posterior summarization in Bayesian phylogenetics using Tracer 1.7. *Syst. Biol*. **67**, 901–904 (2018).

Ronquist, F. *et al*. MrBayes 3.2: Efficient Bayesian phylogenetic inference and model choice across a large model space. *Syst. Biol*. **61**, 539–542 (2012).

Scotese, C. R. *The PALEOMAP Project PaleoAtlas for ArcGIS. Cenozoic Paleogeographic and Plate Tectonic Reconstructions, Vol. 1* (PALEOMAP Project, 2013).

Setiyabudi, E. An Early Pleistocene giant tortoise (Reptilia; Testudines; Testudinidae) from the Bumiayu area, Central Java, Indonesia. *J. Fossil Res*. **42**, 1–11 (2009).

Shaffer, H. B., McCartney-Melstad, E., Near, T. J., Mount, G. G. & Spinks, P. Q. Phylogenomic analyses of 539 highly informative loci dates a fully resolved time tree for the major clades of living turtles (Testudines). *Mol. Phylogenet. Evol*. **115**, 7–15 (2017).

Stamatakis, A. RAxML version 8: a tool for phylogenetic analysis and post-analysis of large phylogenies. *Bioinformatics* **30**, 1312–1313 (2014).

Sukhanov, V. B. Mesozoic turtles of middle and central Asia in *The Age of Dinosaurs in Russia and Mongolia* (eds Benton, M. J., Shishkin, M. A., Unwin, D. M. & Kurochkin, E. N.), 309–367 (Cambridge University Press, 2000).

TEWG [Turtle Extinctions Working Group]. Turtles and tortoises of the world during the rise and global spread of humanity: first checklist and review of extinct Pleistocene and Holocene chelonians. *Chelon. Res. Monogr*. **5**, 1–66 (2015).

TTWG [Turtle Taxonomy Working Group]. *Turtles of the World: Annotated Checklist and Atlas of Taxonomy, Synonymy, Distribution, and Conservation Status (8th Ed.)* (Chelonian Research Foundation and Turtle Conservancy, Chelonian Research Monographs 7, 2017).

Vlachos, E. A review of the fossil record of North American turtles of the clade *Pan-Testudinoidea*. *Bull. Peabody Mus. Nat. Hist*. **59**, 3–94 (2018).

Vlachos, E. & Rabi, M. Total evidence analysis and body size evolution of extant and extinct tortoises (Testudines: Cryptodira: Pan-Testudinidae). *Cladistics* **34**, 652–683 (2018).

Xiong, L., Wang, J. & Nie, L. The complete mitochondrial genome of the Madagascan plowshare tortoise *Astrochelys yniphora* (Testudines, Testudinidae). *Mitochondrial DNA Part B* **4**, 1778–1779 (2019).

Yu, Y., Harris, A. J., Blair, C. & He, X. J. RASP (Reconstruct Ancestral State in Phylogenies): a tool for historical biogeography. *Mol. Phylogenet. Evol*. **87**, 46–49 (2015).
